# Target trial emulation of sodium glucose cotransporter 2 inhibitors and clinical outcomes in diabetes and end stage kidney disease

**DOI:** 10.1038/s41598-026-49221-8

**Published:** 2026-05-05

**Authors:** Jian-Yu Jhu, Yu-Wei Fang, Yueh Chien Lin, Hung-Hsiang Liou, Meng-Ting Chen, Han-Ru Tan, Ming-Hsien Tsai

**Affiliations:** 1 Division of Endocrinology, Department of Internal Medicine, Shin Kong Wu Ho-Su Memorial Hospital, Taipei, Taiwan; 2 Division of Nephrology, Department of Internal Medicine, Shin Kong Wu Ho-Su Memorial Hospital, Taipei, Taiwan; 3 Department of Medicine, Fu Jen Catholic University, New Taipei City, Taiwan; 4 Division of Nephrology, Department of Internal Medicine, Hsin-Jen Hospital, New Taipei City, Taiwan; 5 Data Analytics Team, Department of Digital Medicine, Shin Kong Wu Ho-Su Memorial Hospital, Taipei, Taiwan; 6 Department of Healthcare Management, National Taipei University of Nursing and Health Sciences, Taipei, Taiwan

**Keywords:** Sodium–glucose cotransporter-2 inhibitor, Dipeptidyl peptidase-4 inhibitor, End stage of kidney disease, Mortality, Sepsis, Hospitalization, Type 2 diabetes mellitus, Real-world evidence, Target trial emulation, Diseases, Endocrinology, Medical research, Nephrology

## Abstract

Patients with end-stage kidney disease (ESKD) have been largely excluded from randomized trials of sodium–glucose cotransporter-2 inhibitors (SGLT2is). Despite the lack of guideline recommendations, SGLT2i prescriptions occur in real-world clinical practice. We aimed to describe real-world associations between SGLT2i exposure and clinical outcomes among patients with type 2 diabetes mellitus (T2DM) coded with ESKD. We conducted a target trail emulation with retrospective, new-user, active-comparator cohort study using the TriNetX US Collaborative Network (2016–2023). Adults with T2DM and ESKD who initiated an SGLT2i or a dipeptidyl peptidase-4 inhibitor (DPP4i) were included. Propensity score matching (1:1) was used to balance baseline characteristics. The primary outcome was all-cause mortality; secondary outcomes included sepsis, pneumonia, major adverse cardiovascular events (MACE), all-cause hospitalization, and emergency department visits. Subgroup analyses were exploratory, and heterogeneity was assessed using Cochran’s statistics. After matching, 5295 SGLT2i users were compared with 5295 DPP4i users. Over a follow-up of up to 4 years, SGLT2i exposure was associated with lower all-cause mortality (hazard ratio [HR] 0.90, 95% confidence interval [CI] 0.84–0.97), sepsis (HR 0.87, 95% CI 0.79–0.95), and all-cause hospitalization (HR 0.93, 95% CI 0.89–0.97). No significant associations were observed for MACE, pneumonia, or emergency department visits. Subgroup-specific estimates varied in magnitude, with no consistent evidence of heterogeneity. In this large real-world cohort of patients coded with ESKD, SGLT2i exposure was associated with favorable outcome patterns compared with DPP4i. Given the observational design, potential misclassification of kidney disease status, and off-label drug use, these findings should be interpreted as hypothesis-generating and do not establish causality.

**Supplementary Information:**

The online version contains supplementary material available at 10.1038/s41598-026-49221-8.

## Introduction

Type 2 diabetes mellitus (T2DM) is the leading cause of end-stage kidney disease (ESKD) worldwide and is associated with substantial morbidity, mortality, and health-care utilization^1^. Patients with T2DM and ESKD experience markedly elevated risks of cardiovascular disease, infection, hospitalization, and premature death compared with those without kidney failure^2,3^. Despite advances in renal replacement therapies, clinical outcomes in this population remain poor, highlighting the need to better understand real-world treatment patterns and outcome associations in advanced kidney disease.

Sodium–glucose cotransporter-2 inhibitors (SGLT2is) have transformed the management of T2DM and chronic kidney disease (CKD)^4,5^. Large randomized trials and real-world studies have consistently demonstrated that SGLT2is reduce the risk of CKD progression, heart failure hospitalization, and cardiovascular events across a broad range of kidney function^6,7^. Notably, recent trials such as DAPA-CKD^8^ and EMPA-KIDNEY^9^ extended these benefits to patients with advanced CKD, including those with estimated glomerular filtration rates as low as 20–25 mL/min/1.73 m^2^. However, patients with ESKD—particularly those receiving maintenance dialysis—have been systematically excluded from randomized controlled trials, leaving substantial uncertainty regarding the clinical implications of SGLT2i exposure in this population.

Although current clinical guidelines do not recommend routine SGLT2i use in ESKD, prescriptions occur in real-world clinical practice. This may reflect clinician extrapolation of cardiovascular benefits observed in earlier stages of CKD, as well as emerging mechanistic evidence suggesting potential pleiotropic effects of SGLT2is that are independent of glucose lowering^10^. Experimental and clinical studies have proposed potential pathways through which SGLT2is may influence cardiovascular and systemic outcomes in advanced kidney disease, including modulation of myocardial metabolism, anti-inflammatory effects, improvement in anemia, and preservation of residual kidney function^11–14^. Nevertheless, existing evidence in ESKD remains limited and is largely derived from small observational studies, underscoring the need for cautious interpretation.

Real-world data provide an opportunity to describe prescribing patterns and outcome associations in populations underrepresented in randomized trials, while acknowledging inherent limitations such as confounding and disease misclassification^15^. In large electronic health record databases, patients coded with ESKD represent a heterogeneous population that may include individuals receiving dialysis as well as those with advanced kidney failure approaching dialysis. Examining outcome patterns in this setting is therefore important for hypothesis generation.

Accordingly, we conducted a large multicenter real-world observational study using a federated electronic health record network to evaluate associations between SGLT2i exposure and clinical outcomes among patients with T2DM coded with ESKD. Using a new-user, active-comparator design with propensity score matching, we aimed to describe real-world patterns of mortality, infection-related outcomes, and health-care utilization in an explicitly exploratory analysis.

## Methods

### Data source

TriNetX is a health-care platform that connects hospitals, pharmaceutical companies, and researchers with the goal of improving medical research and patient care^16^. TriNetX provides real-world evidence by analyzing structured electronic health record (EHR) data—including demographics, diagnoses, procedures, prescriptions, labs, and vital signs—from more than 220 healthcare organizations in over 30 countries, primarily academic centers and community hospitals. Advanced analytical and visualization tools enable comprehensive exploration of clinical trends, treatments, and patient outcomes across diverse populations. The platform protects patient privacy through rigorous deidentification processes and provides only aggregated data and summary statistics. This commitment to data security, combined with access to extensive real-world clinical information, has established it as a trusted and widely utilized resource for medical research, clinical decision-making, and healthcare innovation.

### Ethics statement

The study protocol was approved by the Institutional Review Board of Shin Kong Wu Ho-Su Memorial Hospital, Taiwan (approval number: 20240812R), and all methods were performed in accordance with the relevant guidelines and regulations and with the Declaration of Helsinki. The requirement for informed consent was waived because the TriNetX database provides deidentified patient data.

### Study design and cohort

This was a retrospective, new-user, active-comparator cohort study with an intention-to-treat analysis and propensity score matching (PSM) based on target trial emulation guidelines^17^ (see Table S1 for details). We assessed the clinical outcomes in patients with T2DM plus ESKD from 68 health-care organizations in the TriNetX US Collaborative Network. The International Classification of Diseases, 10th Revision code N18.6 is used to classify ESKD, which represents the most severe stage of chronic kidney disease with glomerular filtrate rate less than 15 mL/min/1.73 m^2^, typically requiring renal replacement therapy such as dialysis or kidney transplantation to sustain life. Dipeptidyl peptidase-4 inhibitors (DPP4is) were selected as the active comparator because these drugs are widely recommended for glycemic control in patients with T2DM, including those with advanced CKD^18^. Thus, DPP4is represent a clinically relevant benchmark for evaluating the efficacy and safety of SGLT2is in patients with T2DM and ESKD.

**Fig. 1:**
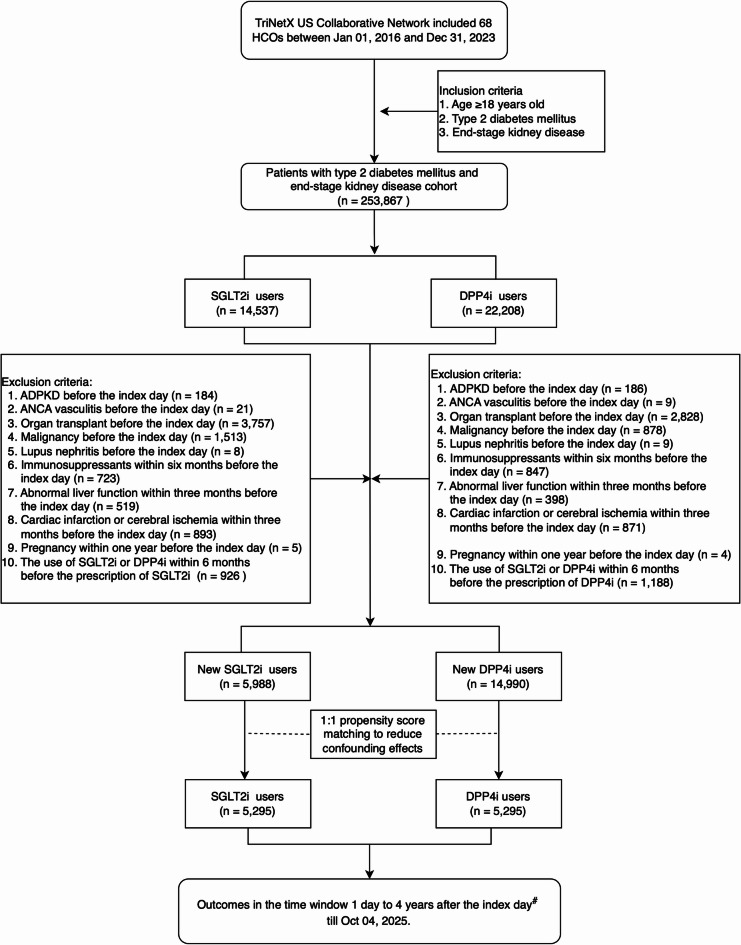
Cohort selection flow diagram showing inclusion and exclusion of patients with type 2 diabetes coded with end-stage kidney disease. HOC, Healthcare Organizations; ESKD, end-stage renaldisease; SGLT2i, sodium–glucose cotransporter 2 inhibitor; DPP4i,dipeptidyl peptidase-4 inhibitor; ADPKD, autosomal dominant polycystickidney disease; ANCA, antineutrophil cytoplasmic antibody.

Figure 1 presents a flowchart depicting patient selection. From the TriNetX database, we identified adult patients (aged ≥ 18 years) with T2DM plus ESKD during the study period—January 1, 2016, to December 31, 2023 (*n* = 253,867). The index date was defined as the date of the first prescription of SGLT2is or DPP4is during the study period plus 90 days. We applied a 90-day lag between treatment initiation and the index date to allow treatment stabilization and reduce reverse causation, whereby early events or deaths may reflect baseline severity rather than medication effects. Patients were classified as SGLT2i users (*n* = 14,537) or DPP4i users (*n* = 22,208) based on their index prescription. Group assignments were maintained regardless of subsequent medication changes, consistent with an intention-to-treat approach. Exclusion criteria included autosomal dominant polycystic kidney disease, ANCA-associated vasculitis, organ transplantation, malignancy, and lupus nephritis prior to the index date, as well as immunosuppressant use within 6 months, abnormal liver function, myocardial infarction, or cerebral ischemia within 3 months, and pregnancy within the past year. Additionally, a 6-month washout period was applied by excluding patients with prior exposure to the study medication before the first prescription. After exclusions, new users were identified: 5988 for SGLT2i and 14,990 for DPP4i. SGLT2i exposure included canagliflozin, dapagliflozin, empagliflozin, ertugliflozin, bexagliflozin, and sotagliflozin. DPP4i exposure included sitagliptin, vildagliptin, saxagliptin, linagliptin, and alogliptin.

To reduce the effects of potential confounders and simulate the comparability of randomized controlled trials, we performed 1:1 PSM, thereby establishing well-matched groups. The study outcomes were assessed at 1 day and up to 4 years after the index date, with all outcomes assessed up through October 4, 2025. Figure S1 presents the study design schematic. This study followed the Strengthening the Reporting of Observational Studies in Epidemiology reporting guidelines for observational studies^19^. Cohort definitions are detailed in Method S1.

### Baseline covariates

Baseline covariates were prespecified and obtained from EHR data within one year before the index date. The analysis included demographic variables (age at index, sex, body mass index (BMI), and race categories), a comprehensive set of comorbidities (dyslipidemia; hypertension; cardiomyopathy; ischemic heart disease; heart failure; aortic aneurysm/dissection; arterial embolism/thrombosis; atrioventricular or left bundle‑branch block; peripheral vascular disease; chronic rheumatic heart disease; pulmonary heart disease; chronic obstructive pulmonary disease; cerebrovascular disease; gout; nicotine dependence; anxiety disorders; thyroid disorders; liver cirrhosis; and fatty liver), medication history (insulin, sulfonylureas, thiazolidinediones, statins, fibrates, renin–angiotensin system blockade (RASB), beta‑blockers, calcium channel blockers, peripheral vasodilators, and antithrombotic agents), and laboratory measures (serum creatinine, blood urea nitrogen, sodium, potassium, calcium, phosphate, magnesium, hemoglobin, alanine aminotransferase, aspartate aminotransferase, alkaline phosphatase, albumin, hemoglobin A1c (HbA1C), low‑density lipoprotein cholesterol, high‑density lipoprotein cholesterol, triglycerides, and C‑reactive protein). The codes for the baseline covariates are shown in Method S1.

### Study outcomes

The primary outcome was the risk of mortality, whereas the secondary outcomes were the risks of sepsis, pneumonia, MACEs, hospitalization, and admission to the emergency department (ED). MACEs include various cardiovascular complications, particularly acute myocardial infarction, heart failure, and stroke. The primary and secondary outcomes were compared between the SGLT2i and DPP4i groups. The outcomes were monitored for a period of 4 years. Outcomes were tracked until the first specified event, death, loss to follow-up, or the study end date (October 04, 2025), whichever occurred first. The outcome codes are detailed in Method S1.

### Sensitivity analysis

To assess the robustness of our findings, we conducted seven prespecified sensitivity analyses. First, we applied alternative propensity score matching (PSM) models incorporating different sets of covariates to evaluate the stability of the estimated associations and reduce residual confounding. Second, we examined outcome associations across predefined follow‑up intervals (1 day to 1 year, 2 years, and 3 years) to address potential violations of the proportional hazards assumption and to assess the temporal consistency of the results. Third, we repeated the analyses without applying a 3‑month post‑initiation lag period, thereby capturing early events after treatment initiation and evaluating the impact of delayed observation. Fourth, we evaluated clinical outcomes using three alternative definitions of ESKD: estimated glomerular filtration rate < 15 mL/min/1.73 m^2^, < 10 mL/min/1.73 m^2^, or dialysis dependence. Fifth, we restricted analyses to data from 2000 to 2023 to address time-period bias. Sixth, to mitigate immortal time bias related to the lag period, we excluded patients who died before the lag ended (90 days after the first prescription of study medications). Finally, we added more antidiabetic medications (glucagon-like peptide-1 receptor agonists and repaglinide) to the propensity score matching.

### Statistical analysis

Data are presented as number (percentage) values for categorical variables and mean ± standard deviation (SD) values for continuous variables. Standardized mean difference (SMD) values were calculated to determine between-group differences, with values of < 0.2, 0.2–0.49, and 0.5–0.8 indicating small, moderate, and large effects, respectively^20^. PSM was performed to minimize baseline confounding and ensure between-group similarity for accurate treatment effect comparisons. Baseline parameters with SMD values of < 0.1 indicating well-balanced groups^21^. Propensity scores were generated using logistic regression and matched by greedy nearest-neighbor matching with a 0.1 caliper to ensure balanced treatment groups for analysis. During PSM, age on the index date was handled as a continuous variable, while sex, race, lifestyle factors, comorbidities, and medication use were treated as categorical (present/absent). Laboratory parameters were considered present if available or absent if missing within the specified time window (see Method S2 for details).

A Cox proportional-hazards regression model was used to compare the outcomes between the SGLT2i and DPP4i groups. The proportional-hazards assumption was assessed using the generalized Schoenfeld approach integrated into the TriNetX platform. Kaplan–Meier curves with log-rank test were generated to estimate event-free probabilities. The E-value was computed for each outcome to quantify the minimum strength of association that an unmeasured confounder would need to have, with both the exposure and the outcome, to fully explain the observed effect estimate, beyond the measured covariates^22^.

Subgroup analyses were conducted, with all subgroups rematched via PSM on baseline variables to ensure well-balanced, comparable pairs. Because the interaction effect was not supported on the TriNetX platform, we assessed subgroup heterogeneity via Cochran’s Q test and I^2^ statistics under a random-effects model^23^. The p-value for heterogeneity was computed to ascertain whether the impact of SGLT2i on clinical outcomes exhibited significant variation across subgroups.

All the statistical analyses were performed on the TriNetX platform. A two-sided *P* value of < 0.05 indicated statistical significance. Figures and heterogeneity p-values were generated using R (version 4.4.2; R Foundation for Statistical Computing, Vienna, Austria).

## Results

### Baseline characteristics

Table 1 presents the baseline characteristics of patients receiving SGLT2i (*n* = 5988) versus DPP4i (*n* = 14,990) before matching, and after propensity score matching (*n* = 5295 per group). Before matching, the SGLT2i group showed notable differences including younger age (63 vs. 64.5 years), higher BMI (33.2 vs. 31.3 kg/m^2^), greater prevalence of dyslipidemia (82.1% vs. 69.9%), cardiovascular comorbidities including cardiomyopathy (18.6% vs. 10.3%) and heart failure (47.9% vs. 38.8%), and greater use of RASB (71.8% vs. 56.3%). The laboratory values revealed lower creatinine (3.0 vs. 4.7 mg/dL) and urea nitrogen (42.3 vs. 51.6 mg/dL) but higher hemoglobin (11.3 vs. 10.4 g/dL) and HbA1c (7.7% vs. 7.3%) in the SGLT2i group. After PSM incorporating all baseline parameters, both cohorts achieved excellent balance with all standardized mean differences less than 0.1, indicating negligible between-group differences and ensuring comparable populations for subsequent outcome analyses.

**Table 1:** Baseline characteristics of patients with type 2 diabetes coded with end-stage kidney disease.

Parameters	Before matching			After matching		
	SGLT2i (*n* = 5,988)	DPP4i (*n* = 14,990)	SMD^#^	SGLT2i (*n* = 5,295)	DPP4i (*n* = 5,295)	SMD^#^
Age at Index (years), mean ± SD	63 ± 11.7	64.5 ± 11.6	0.125	63.4 ± 11.6	63.7 ± 12	0.020
Male, *n* (%)	3419 (57.1)	8076 (53.9)	0.065	2971 (56.1)	2920 (55.1)	0.019
BMI (kg/m^2^), mean ± SD	33.2 ± 8	31.3 ± 7.6	0.247	32.8 ± 7.9	32.7 ± 8	0.011
Race, *n* (%)						
White people	3152 (52.6)	6602 (44)	0.173	2737 (51.7)	2750 (51.9)	0.005
African American	1530 (25.6)	4302 (28.7)	0.071	1367 (25.8)	1367 (25.8)	< 0.001
Asian	363 (6.1)	1438 (9.6)	0.132	341 (6.4)	336 (6.3)	0.004
Other race	314 (5.2)	983 (6.6)	0.056	285 (5.4)	281 (5.3)	0.003
Comorbidity, *n* (%)						
Dyslipidemia	4915 (82.1)	10,481 (69.9)	0.288	4271 (80.7)	4268 (80.6)	0.001
Hypertensive diseases	5622 (93.9)	13,818 (92.2)	0.067	4960 (93.7)	4962 (93.7)	0.002
Cardiomyopathy	1111 (18.6)	1542 (10.3)	0.237	858 (16.2)	828 (15.6)	0.015
Ischemic heart diseases	2856 (47.7)	6017 (40.1)	0.153	2439 (46.1)	2430 (45.9)	0.003
Heart failure	2869 (47.9)	5816 (38.8)	0.185	2439 (46.1)	2407 (45.5)	0.012
Aortic aneurysm and dissection	177 (3)	284 (1.9)	0.069	138 (2.6)	138 (2.6)	< 0.001
Arterial embolism and thrombosis	126 (2.1)	163 (1.1)	0.081	108 (2)	94 (1.8)	0.019
AV and left bundle-branch block	729 (12.2)	1279 (8.5)	0.120	605 (11.4)	609 (11.5)	0.002
PVD	463 (7.7)	932 (6.2)	0.059	403 (7.6)	400 (7.6)	0.002
Chronic rheumatic heart diseases	758 (12.7)	1295 (8.6)	0.131	621 (11.7)	632 (11.9)	0.006
Pulmonary heart disease	1124 (18.8)	1904 (12.7)	0.167	925 (17.5)	929 (17.5)	0.002
COPD	971 (16.2)	1925 (12.8)	0.096	835 (15.8)	840 (15.9)	0.003
Cerebrovascular diseases	943 (15.7)	2181 (14.6)	0.033	846 (16)	826 (15.6)	0.010
Gout	866 (14.5)	1855 (12.4)	0.061	738 (13.9)	739 (14)	0.001
Nicotine dependence	888 (14.8)	1601 (10.7)	0.125	747 (14.1)	771 (14.6)	0.013
Other anxiety disorders	1234 (20.6)	1946 (13)	0.205	1018 (19.2)	1012 (19.1)	0.003
Disorders of thyroid gland	1368 (22.8)	2911 (19.4)	0.084	1205 (22.8)	1229 (23.2)	0.011
Liver cirrhosis	348 (5.8)	589 (3.9)	0.088	292 (5.5)	300 (5.7)	0.007
Fatty liver	446 (7.4)	478 (3.2)	0.191	326 (6.2)	324 (6.1)	0.002
Medication, *n* (%)						
Insulin	4425 (73.9)	10,317 (68.8)	0.112	3877 (73.2)	3897 (73.6)	0.009
Sulfonylureas	1285 (21.5)	4210 (28.1)	0.154	1227 (23.2)	1302 (24.6)	0.033
Thiazolidinediones	280 (4.7)	722 (4.8)	0.007	252 (4.8)	263 (5)	0.010
Statin	4724 (78.9)	11,181 (74.6)	0.102	4143 (78.2)	4161 (78.6)	0.008
Fibrates	420 (7)	832 (5.6)	0.060	367 (6.9)	371 (7)	0.003
RASB	4301 (71.8)	8441 (56.3)	0.328	3682 (69.5)	3767 (71.1)	0.035
Beta-blocker	4380 (73.1)	11,148 (74.4)	0.028	3877 (73.2)	3867 (73)	0.004
Calcium channel blockers	3466 (57.9)	9752 (65.1)	0.148	3160 (59.7)	3196 (60.4)	0.014
Peripheral vasodilator	145 (2.4)	438 (2.9)	0.031	134 (2.5)	143 (2.7)	0.011
Antithrombotic agents	4180 (69.8)	10,543 (70.3)	0.012	3695 (69.8)	3699 (69.9)	0.002
Laboratory, mean ± SD						
Creatinine (mg/dL)	3 ± 4.4	4.7 ± 3.4	0.423	3.2 ± 4.6	3.4 ± 3.2	0.034
Urea nitrogen (mg/dL)	42.3 ± 26.4	51.6 ± 27.9	0.343	43.7 ± 26.8	45.1 ± 26.8	0.054
Sodium(mmol/L)	137.9 ± 3.9	137.6 ± 4.1	0.071	137.9 ± 3.9	137.9 ± 4.1	0.019
Potassium(mmol/L)	4.4 ± 0.6	4.4 ± 0.7	0.102	4.4 ± 0.6	4.4 ± 0.6	0.020
Calcium (mg/dL)	9 ± 0.8	8.8 ± 0.8	0.212	9 ± 0.8	8.9 ± 0.8	0.046
Phosphate (mg/dL)	4.2 ± 1.4	4.2 ± 1.5	0.254	4.3 ± 1.4	4.3 ± 1.4	0.028
Magnesium (mg/dL)	2 ± 0.4	2 ± 0.4	0.114	2 ± 0.4	2 ± 0.4	0.016
Hemoglobin (g/dL)	11.3 ± 2.4	10.4 ± 2.1	0.410	11.1 ± 2.4	10.9 ± 2.2	0.088
ALT (U/L)	25 ± 81.5	23.7 ± 63.9	0.017	23.1 ± 30.1	26.6 ± 90.2	0.053
AST(U/L)	28.1 ± 155.3	26.9 ± 103.2	0.009	25.7 ± 44.4	31.5 ± 159.5	0.050
Alkaline phosphatase (U/L)	106.5 ± 94.9	106.9 ± 73.6	0.004	106.5 ± 94.7	106.8 ± 82.2	0.003
Albumin (g/dL)	3.6 ± 0.7	3.5 ± 0.7	0.143	3.6 ± 0.7	3.6 ± 0.7	0.083
Hemoglobin A1c (%)	7.7 ± 1.9	7.3 ± 1.8	0.215	7.6 ± 1.9	7.6 ± 1.9	0.033
LDL (mg/dL)	82.2 ± 43.1	82.8 ± 42.2	0.015	82.4 ± 43.3	83.2 ± 42.7	0.020
HDL (mg/dL)	41.2 ± 16.6	40.9 ± 17.9	0.020	41.3 ± 16.6	40.4 ± 17.8	0.055
Triglyceride (mg/dL)	180.1 ± 158.2	168.9 ± 152.4	0.072	179 ± 158.2	174.9 ± 177.5	0.025
C reactive protein (mg/dL)	4.2 ± 1.4	4.6 ± 1.5	0.254	4.3 ± 1.4	4.3 ± 1.4	0.028

^&^The propensity score matching includes all parameters in this table to achieve balanced cohorts.

^#^A SMD value below 0.1 indicates a negligible difference between groups.

SGLT2i, sodium-glucose co-transporter 2 Inhibitors; DPP4i, dipeptidyl peptidase-4 inhibitors ; BMI, body mass index; PVD, peripheral vascular disease; COPD, chronic obstructive pulmonary disease; RASB, renin-angiotensin system blockade; ALT, aspartate aminotransferase ; AST, aspartate aminotransferase; LDL, low density lipoprotein; HDL, high density lipoprotein.

### All-cause mortality effect of SGLT2i in ESKD

**Fig. 2:**
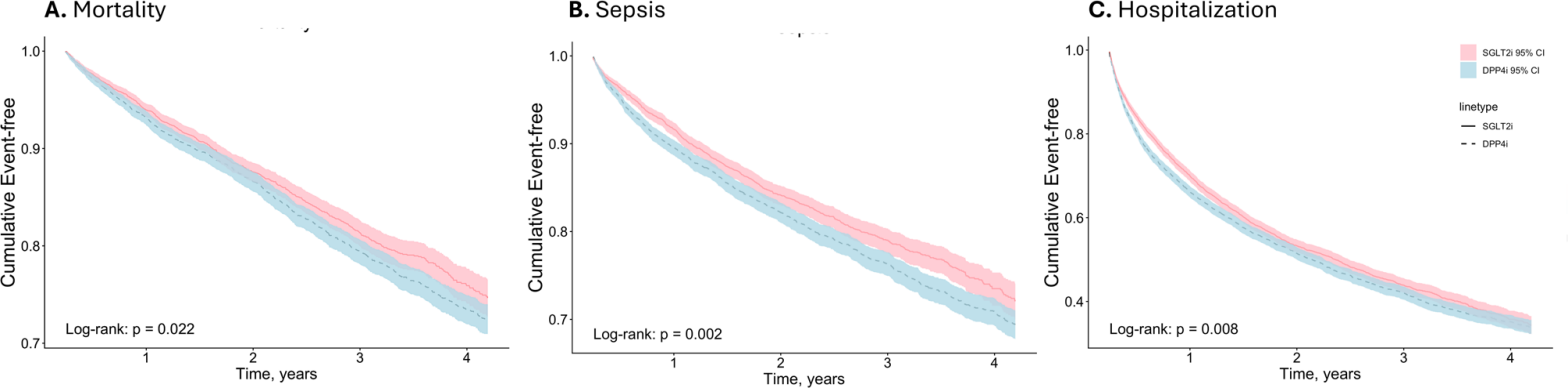
Kaplan–Meier curves among propensity score–matched patients initiating SGLT2i or DPP-4i for (**A**) Mortality. (**B**) Sepsis. (**C**) Hospitalization. SGLT2i, sodium–glucose cotransporter 2 inhibitor; DPP4i,dipeptidyl peptidase-4 inhibitor.

Figure S2 shows the follow-up time distribution. The mean follow-up time was 817 ± 442 days for the SGLT2i group and 950 ± 520 days for the DPP4i group. The Kaplan–Meier curve presented in Fig. 2A indicates a greater event-free probability for all-cause mortality in the SGLT2i group than in the DPP4i group (log-rank *P* = 0.022). Throughout the follow-up period, the incidence of adverse outcomes was consistently lower in the SGLT2i group than in the DPP4i group, with the curves diverging over time.

In the propensity score–matched cohort, all‑cause mortality occurred less frequently among patients initiating SGLT2is than among those initiating DPP4is (Table 2). Over a follow‑up period of up to four years, the cumulative incidence of mortality was 25.32% in the SGLT2i group and 27.49% in the DPP4i group. SGLT2i exposure was associated with a lower risk of all‑cause mortality (hazard ratio [HR] 0.90; 95% confidence interval [CI] 0.82–0.98). The E-value for the point estimate was 1.46, indicating that an unmeasured confounder would need to be associated with both SGLT2i exposure and all-cause mortality by a risk ratio of at least 1.46 (46% higher risk) to fully explain away the observed effect. The E-value for the lower confidence limit was 1.74, suggesting the association between SGLT2i use and lower mortality is moderately robust to unmeasured confounding.

**Table 2:** Associations between SGLT2i exposure and clinical outcomes compared with DPP-4i in propensity score-matched patients.

Clinical Outcomes	After propensity score matching^%^ with a follow-up of 4 years							
	SGLT2i users (*n* = 5,295 )		DPP4i users (*n* = 5,295 )		SGLT2i vs. DPP4i		E-value	
	Events (*n*)	Cumulative incidence (%)	Events (*n*)	Cumulative incidence (%)	HR (95% CI)	*P* value	Point estimate	Lower 95% CI
Primary outcomes								
Mortality	784	25.3	1021	27.5	0.90 (0.82–0.98)	0.022	1.46	1.74
Secondary outcomes								
Sepsis	881	27.8	1119	30.6	0.87 (0.80–0.95)	0.002	1.56	1.81
Pneumonia	790	26.2	937	26.4	0.95 (0.86–1.04)	0.281	1.29	1.00
MACE^#^	1116	33.5	1235	32.9	1.00 (0,92–1.08)	0.99	1.00	1.00
Hospitalization	2421	65.6	2675	66.1	0.93 (0.88–0.98)	0.008^†^	1.36	1.53
ED visiting	2138	59.3	2253	57.8	1.02 (0.96–1.09)	0.448	1.16	1.00

^%^Propensity score matching used all the baseline variable in Table 1.

^#^MACE included the acute myocardial infarction, heart failure, stroke, and death.

^†^The proportional hazards assumption is violated (*p* < 0.05).

SGLT2i, sodium-glucose cotransporter-2 inhibitor; DPP4i, dipeptidyl peptidase-4 inhibitors; HR, hazard ratio; CI, confidence interval; MACE, major adverse cardiovascular events; ED, emergency department.

### Other clinical outcomes effect of SGLT2i in ESKD

Kaplan–Meier analyses demonstrated higher event‑free probabilities for sepsis (Fig. 2B) and all‑cause hospitalization (Fig. 2C) among patients initiating SGLT2i compared with those initiating DPP4i (log‑rank *P* = 0.002 and 0.008, respectively). In the propensity score–matched cohort, SGLT2i exposure was associated with a lower cumulative incidence of sepsis (27.8% vs. 30.6%; HR 0.87, 95% CI 0.80–0.95) and all‑cause hospitalization (65.6% vs. 66.1%; HR 0.93, 95% CI 0.88–0.98) compared with DPP4i exposure.

For secondary outcomes, the E-value for sepsis was 1.56 (lower confidence limit: 1.81), indicating that an unmeasured confounder would need to increase the risk of both SGLT2i exposure and sepsis by at least 56% to nullify the observed association, suggesting moderate robustness to unmeasured confounding. For all-cause hospitalization, the E-value was 1.36 (lower confidence limit: 1.53), implying that a 36% increased risk association with both exposure and outcome would be required to explain away the findings. In contrast, no statistically significant associations were observed for pneumonia, major adverse cardiovascular events, or emergency department visits (Table 2). Kaplan–Meier curves for these outcomes are presented in Fig. S3.

### Results of subgroup analysis

**Fig. 3:**
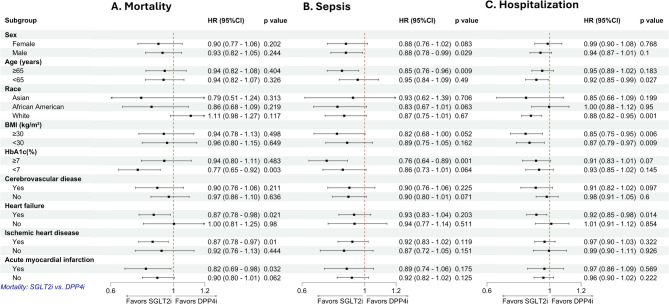
Results of a subgroup analysis for the outcomes of (**A**) Mortality. (**B**) Sepsis. (**C**) Hospitalization. The vertical line indicates an HR of 1.00; A 95% CI value with a lower limit of > 1.00 indicates a higher risk of events. SGLT2i, sodium–glucose cotransporter 2 inhibitor; DPP4i,dipeptidyl peptidase-4 inhibitor; HR, hazard ratio; CI, confidence interval;BMI, body mass index; HbA1c, glycated hemoglobin.

In subgroup analyses of all‑cause mortality (Fig. 3A), the direction of association between SGLT2i exposure and mortality was generally consistent across most prespecified subgroups. Statistically significant associations were observed among patients with heart failure (HR 0.87, 95% CI 0.78–0.98), ischemic heart disease (HR 0.87, 95% CI 0.78–0.97), and prior acute myocardial infarction (HR 0.82, 95% CI 0.69–0.98). Among male patients, the estimate suggested a similar direction of association, although statistical significance was not reached (HR 0.93, 95% CI 0.82–1.05).

For sepsis (Fig. 3B), SGLT2 inhibitor exposure was associated with lower risks in selected subgroups, including patients younger than 65 years (HR 0.85, 95% CI 0.76–0.96), those with a body mass index ≥ 30 kg/ m^2^ (HR 0.82, 95% CI 0.68–1.00), and those with baseline HbA1c ≤ 7% (HR 0.76, 95% CI 0.64–0.89). In analyses of all‑cause hospitalization (Fig. 3C), statistically significant associations were observed in patients with body mass index ≥ 30 kg/ m^2^ (HR 0.85, 95% CI 0.75–0.95) as well as those with body mass index < 30 kg/m^2^ (HR 0.87, 95% CI 0.79–0.97).

Overall, subgroup‑specific estimates were largely directionally consistent, with no evidence of statistically significant heterogeneity across subgroups. Detailed outcome event distributions are provided in Figs. S4–S6 for reference.

### Results of the sensitivity analysis

Sensitivity analyses demonstrated consistent findings across multiple propensity score matching specifications with extensive covariate adjustment (Table S2). Results were also stable across all prespecified follow-up intervals (Table S3) and were not materially altered when analyses were conducted without a 3-month post-initiation lag period (Table S4). In addition, analyses applying alternative definitions of advanced kidney disease yielded results consistent with the primary analysis (Table S5). Furthermore, results were robust when using 2000–2023 data to address time bias (Table S6), excluding all-cause mortality during follow-up to mitigate immortal time bias (Table S7), and adding additional antidiabetic medications for ESKD to the propensity score matching (Table S8).

### Results of adverse effects analysis

After propensity score matching (4835 patients in each group), no statistically significant differences were observed between groups with respect to adverse events (Table S9). The incidence of genital infections was 18.2% among SGLT2 inhibitor users and 17.7% among DPP-4 inhibitor users; ketoacidosis occurred in 3.1% and 2.5%, respectively; and hypoglycemia was reported in 11.8% and 11.4%, respectively. Overall, the two treatment groups exhibited similar safety profiles within this high-risk population.

## Discussion

In this large multicenter observational study using real world data, we evaluated clinical outcomes associated with SGLT2 inhibitor exposure among patients with type 2 diabetes coded with end stage kidney disease. Although SGLT2i are not currently recommended for patients with ESKD, their use occurs in routine clinical practice. In this context, SGLT2i exposure was associated with lower risks of all-cause mortality, sepsis, and hospitalization compared with DPP4i. These findings should be interpreted cautiously, as the study was not designed to establish causality and is subject to the inherent limitations of observational data.

Several considerations are essential when interpreting these findings. First, classification of ESKD in administrative databases may encompass a heterogeneous population, including individuals receiving dialysis as well as those with advanced kidney failure approaching dialysis initiation. Second, important clinical variables such as dialysis modality, dialysis vintage, and residual kidney function were not available in the data source. Consequently, residual confounding and exposure misclassification cannot be excluded, and the observed associations should be regarded as exploratory.

SGLT2i has emerged as a promising treatment option for patients with advanced CKD, conferring considerable renal and cardiovascular benefits regardless of T2DM status in two randomized trials of DAPA-CKD and EMPA-CKD^8,9^. Originally developed for glycemic control in patients with T2DM, SGLT2is have proven effective in decelerating the progression of CKD and reducing the risks of MACEs and hospitalization for heart failure and cardiovascular death in real-world studies^24,25^. In a target trial emulation using Taiwan’s NHIRD, initiating SGLT2i in patients with T2DM and stage 5 CKD was linked to significantly lower risks of dialysis, cardiovascular events, diabetic ketoacidosis, and acute kidney injury than nonuse, without a difference in all-cause mortality^26^. These findings have highlighted their dual role in renal protection and cardiovascular support. Although evidence supporting SGLT2i continues to grow, long-term trials are needed to confirm their lasting benefits and clarify their safety in advanced CKD patients, especially those without T2DM^27^. Nevertheless, they are now recognized as a key therapies for CKD management, providing value even in advanced-stage patients.

SGLT2i has historically been reserved for patients with CKD not yet undergoing maintained dialysis. However, recent evidence suggests that initiating SGLT2is at the start of dialysis under any conditions may be advantageous. Wang et al. reported that patients with T2DM who were started on SGLT2is at the initiation of dialysis exhibited marked reductions in the risks of MACE and all-cause mortality, with adjusted HRs of 0.52 (95%CI 0.36–0.75) and 0.49 (95%CI 0.34–0.69), respectively, compared with the risks in nonusers at the 5-year follow-up^28^. In a small cohort of patients on incremental hemodialysis (*n* = 7), SGLT2i use was associated with preservation of residual kidney function, demonstrated by improved residual kidney urea clearance over 12 months—a factor linked to enhanced survival and cardiovascular outcomes—alongside reductions in HbA1c, blood pressure, proteinuria, serum uric acid, and interdialytic weight gain^29^. Similarly, a single-center retrospective study of peritoneal dialysis patients (*n* = 16) demonstrated that six months of SGLT2i therapy maintained residual kidney function and urine output in both diabetic and nondiabetic individuals, with minimal adverse events and trends toward lower proteinuria and blood pressure^30^. It has been hypothesized that SGLT2is may benefit patients undergoing peritoneal dialysis by preserving residual kidney function and protecting the peritoneal membrane, thereby reducing the likelihood of transition to hemodialysis^31,32^. However, current evidence, mostly from low-quality observational studies, highlights the need for large randomized controlled trials to guide treatment. Careful monitoring and individualized care are essential to reduce risks in ESKD patients.

In our study, the identification of more than 5,000 SGLT2i users among patients with ESKD reflects a growing real-world trend of off-label SGLT2i use in this population, despite traditional contraindications. This emerging practice aligns with accumulating evidence suggesting that the benefits of SGLT2 inhibitors extend beyond glycemic control, particularly in reducing cardiovascular risk. Major cardiovascular outcome trials, such as EMPA-REG OUTCOME^33^ and DAPA-CKD^8^, have demonstrated that SGLT2i is associated with lower risks of heart failure and mortality in patients with chronic kidney disease. Consistent with these findings, our study revealed significant survival benefits associated with SGLT2i use in patients with T2DM and ESKD undergoing dialysis. Subgroup analyses further revealed that this survival advantage was especially pronounced in patients with preexisting cardiovascular disease. Given that cardiovascular disease remains the leading cause of death in ESKD patients, these results suggest that SGLT2i therapy may offer meaningful cardiovascular protection in this high-risk group.

Through multiple mechanisms, SGLT2is might increase the duration of survival and reduce the risks of sepsis and hospitalization in patients undergoing dialysis. First, these drugs confer substantial cardiovascular benefits. SGLT2i induces a metabolic shift in the myocardium from glucose to ketone bodies, thereby boosting cardiac function and mitigating oxidative stress^34,35^. Second, SGLT2is exert anti-inflammatory^36,37^ and vascular-protective effects^38^, thus alleviating systemic inflammation and endothelial dysfunction—factors associated with sepsis and other complications in individuals undergoing dialysis. The anti-inflammatory effects of SGLT2is may bolster immune defenses and reduce susceptibility to severe infection. Finally, SGLT2is may help preserve residual kidney function, improving fluid and toxin management. The favorable safety profile of SGLT2is allows these benefits to be realized without any considerable increase in the risk of adverse events. Thus, these drugs hold promise for improving key clinical outcomes in patients with ESKD.

Although SGLT2i have consistently been shown to provide notable cardiovascular benefits in the earlier stages of CKD^8,33^. Our findings indicate that this advantage does not extend to patients who have already progressed to ESKD, as no significant reduction in MACE was observed in our ESRD cohort. One possible explanation for this outcome is that by the time patients advance to the ESKD stage, the degree of cardiac damage and overall cardiovascular burden has already become extensive, limiting the potential for any further protective effect. At such an advanced stage of disease, initiating treatment with an SGLT2i may therefore occur too late in the course of cardiac decline to offer meaningful clinical benefit. This pattern closely mirrors the finding of statin therapy in the same population, where the intervention also yields no significant cardiovascular improvement once significant end-organ damage has been established^39,40^.

The strengths of our study lie in its large, multicenter design; the use of real-world data from the robust TriNetX database; and Its comprehensive new-user, active-comparator cohort design with PSM, which on the basis of the principle of target trial emulation, closely approximates a randomized controlled trial. Detailed assessment of comorbidities, medication use, and laboratory parameters, along with robust sensitivity analyses, enhanced confidence in our findings. Overall, this study offers crucial real-world evidence that may inform and broaden guideline recommendations for SGLT2i use in ESKD.

However, several limitations should be acknowledged. First, the retrospective observational design and reliance on EHR data preclude causal inference, and residual confounding from unmeasured factors, such as diabetes duration, kidney disease duration, and dialysis vintage, may persist despite excellent covariate balance after 1:1 PSM (SMD < 0.1). Second, because TriNetX does not support advanced target trial emulation methods, such as sequential trial design or clone-censor-weighting, we could not fully address time-related biases, including treatment switching and immortal time bias. Third, reliance on ICD-10 codes and structured EHR documentation introduces the potential for exposure and outcome misclassification. For example, ESKD codes may capture a heterogeneous population ranging from CKD stage 5 to patients receiving chronic dialysis, and the federated database lacks granular dialysis-related information, including modality, dialysis vintage, and residual kidney function. Fourth, although we used a new-user, active-comparator design with a 90-day lag period, the database does not provide sufficiently detailed information on medication duration, persistence, adherence, or dose adjustment; thus, a single-prescription threshold for cohort entry may not fully reflect chronic exposure. Fifth, owing to platform limitations, we used heterogeneity tests rather than formal interaction p-values in subgroup analyses, so these findings should be considered exploratory and require confirmation in future studies. Sixth, SGLT2i use in ESKD represents off-label prescribing, and our findings reflect real-world clinical practice that may not fully align with current labeling or guideline recommendations. Seventh, despite a 180-day washout period, misclassification of prevalent users cannot be completely excluded because medication history before the lookback window is unavailable, which is an inherent limitation of EHR-based databases. Finally, because White patients comprised 52% of the cohort and represented the largest ethnic subgroup in this US-based dataset, the generalizability of our findings to other racial, ethnic, and non-US populations may be limited. Future studies using more diverse, multinational datasets are needed to confirm the broader applicability of these findings.

## Conclusion

This study provides real-world evidence from a target trial emulation framework suggesting that initiation of SGLT2is is associated with lower risks of all-cause mortality, sepsis, and hospitalization among patients with type 2 diabetes mellitus coded with ESKD, with more pronounced associations in those with pre-existing cardiovascular disease. These findings highlight the potential clinical relevance of SGLT2i use in this high-risk population but should be interpreted as hypothesis-generating. Further prospective studies and randomized trials are needed to confirm these associations and to define optimal timing and patient selection in advanced kidney disease.

## Supplementary Information

Below is the link to the electronic supplementary material.

Supplementary Material 1

## Data Availability

TriNetX is a network connecting multiple research centers, offering instant access to anonymized data from the electronic health records of participating health-care organizations. Qualified researchers and health-care professionals can access this comprehensive database through the dedicated online portal at https://live.trinetx.com. This database facilitates observational studies and offers clinical insights without the need for traditional data-sharing agreements between individual institutions.

## Abbreviations

HOC: Healthcare organizations
ESKD: End-stage kidney disease
SGLT2i: Sodium–glucose cotransporter 2 inhibitor
DPP4i: Dipeptidyl peptidase-4 inhibitor
ADPKD: Autosomal dominant polycystic kidney disease
ANCA: Antineutrophil cytoplasmic antibody
