# Supplementary material for: Target trial emulation of sodium glucose cotransporter 2 inhibitors and clinical outcomes in diabetes and end stage kidney disease
Source: Sci Rep. 2026 May 5;16:20712. doi: 10.1038/s41598-026-49221-8 (PMC13333836; doi:10.1038/s41598-026-49221-8)
Supplement: Supplementary file 1 — Supplementary Material 1 [file 41598_2026_49221_MOESM1_ESM.pdf]

Supplementary Online Content

Method S1 Code for Cohorts, outcomes, and baseline data 2
Method S2. The detail of propensity score matching in TriNetX platform 8
Figure S1. Schematic of study design 13
Figure S2. Time distribution of studied cohorts 14
Figure S3. KM for other clinical outcomes 15
Figure S4. Subgroup analysis for mortality 16
Figure S5. Subgroup analysis for sepsis 17
Figure S6. Subgroup analysis for hospitalization 18
Table S1. Target trial emulation 19
Table S2. Hierarchical propensity score matching models 21
Table S3. Different time frames analysis 22
Table S4. Clinical outcomes analysis without time lag post-index 23
Table S5. Outcomes in the different types of end stage of kidney disease 24
Table S6. Clinical outcomes analysis using 2000–2023 data 25
Table S7. Clinical outcomes analysis excluding the death in lag period 26
Table S8. Clinical outcomes analysis including more anti-diabetics medications 27
Table S9. Side effects analysis in the patients with types 2 DM and ESKD 28

Method S1 Code for Cohorts, outcomes, and baseline data

1. Code for Cohort

**Inclusion criteria**		
**Type 2 DM, ESKD, and target medications**		
visit	TNX:Visit	Visit
diagnosis	UMLS:ICD10CM:E11	Type 2 diabetes mellitus (at least 18 years old at event)
diagnosis	UMLS:ICD10CM:N18.6	End stage renal disease
medication	NLM:ATC:A10BK	Sodium-glucose co-transporter 2 (SGLT2) inhibitors
medication	NLM:ATC:A10BH	Dipeptidyl peptidase 4 (DPP-4) inhibitors
**Exclusion criteria**		
diagnosis	UMLS:ICD10CM:Q61.2	Polycystic kidney, adult type
diagnosis	UMLS:ICD10CM:I77.82	Antineutrophilic cytoplasmic antibody [ANCA] vasculitis
diagnosis	UMLS:ICD10CM:Z94	Transplanted organ and tissue status
medication	NLM:VA:IM900	IMMUNOLOGICAL AGENTS,OTHER
medication	NLM:VA:IM700	IMMUNE STIMULANTS
medication	NLM:VA:IM600	IMMUNE SUPPRESSANTS
laboratory	TNX:9050	Bilirubin.total [Mass/volume] in Serum, Plasma or Blood (at least 2.40 mg/dL)
laboratory	TNX:9044	Alanine aminotransferase [Enzymatic activity/volume] in Serum, Plasma or Blood (at least 102.00 U/L)
laboratory	TNX:9047	Aspartate aminotransferase [Enzymatic activity/volume] in Serum or Plasma (at least 99.00 U/L)
diagnosis	UMLS:ICD10CM:C00-C14	Malignant neoplasms of lip, oral cavity and pharynx
diagnosis	UMLS:ICD10CM:C15-C26	Malignant neoplasms of digestive organs
diagnosis	UMLS:ICD10CM:C30-C39	Malignant neoplasms of respiratory and intrathoracic organs
diagnosis	UMLS:ICD10CM:C40-C41	Malignant neoplasms of bone and articular cartilage
diagnosis	UMLS:ICD10CM:C43-C44	Melanoma and other malignant neoplasms of skin
diagnosis	UMLS:ICD10CM:C45-C49	Malignant neoplasms of mesothelial and soft tissue
diagnosis	UMLS:ICD10CM:C50-C50	Malignant neoplasms of breast (C50)

diagnosis	UMLS:ICD10CM:C51-C58	Malignant neoplasms of female genital organs
diagnosis	UMLS:ICD10CM:C60-C63	Malignant neoplasms of male genital organs
diagnosis	UMLS:ICD10CM:C64-C68	Malignant neoplasms of urinary tract
diagnosis	UMLS:ICD10CM:C69-C72	Malignant neoplasms of eye, brain and other parts of central nervous system
diagnosis	UMLS:ICD10CM:C73-C75	Malignant neoplasms of thyroid and other endocrine glands
diagnosis	UMLS:ICD10CM:C76-C80	Malignant neoplasms of ill-defined, other secondary and unspecified sites
diagnosis	UMLS:ICD10CM:C7A-C7A	Malignant neuroendocrine tumors (C7A)
diagnosis	UMLS:ICD10CM:C81-C96	Malignant neoplasms of lymphoid, hematopoietic and related tissue
diagnosis	UMLS:ICD10CM:C7B-C7B	Secondary neuroendocrine tumors (C7B)
diagnosis	UMLS:ICD10CM:I21	Acute myocardial infarction
diagnosis	UMLS:ICD10CM:I63	Cerebral infarction
diagnosis	UMLS:ICD10CM:G45.9	Transient cerebral ischemic attack, unspecified
diagnosis	UMLS:ICD10CM:I20.0	Unstable angina
diagnosis	UMLS:ICD10CM:M32.14	Glomerular disease in systemic lupus erythematosus
diagnosis	UMLS:ICD10CM:Z33.1	Pregnant state, incidental

2. Outcome Definitions

Table below outlines the definitions for each outcome and the analysis specifications. For outcome definitions consisting of more than one term, at least one term must match. Please see Appendix C for the text representation of the outcome definitions.

**MACE**		
**Outcome definition**		
Diagnosis	UMLS:ICD10CM:I21	Acute myocardial infarction
Diagnosis	UMLS:ICD10CM:I63	Cerebral infarction
Diagnosis	UMLS:ICD10CM:I62	Other and unspecified nontraumatic intracranial hemorrhage
Diagnosis	UMLS:ICD10CM:I22	Subsequent ST elevation (STEMI) and non-ST elevation (NSTEMI) myocardial infarction
Diagnosis	UMLS:ICD10CM:I46	Cardiac arrest
**Mortality**		
**Outcome definition**		
Demographics	Deceased	Deceased
**sepsis**		

**Outcome definition**		
Diagnosis	UMLS:ICD10CM:A40	Streptococcal sepsis
Diagnosis	UMLS:ICD10CM:A41	Other sepsis
Diagnosis	UMLS:ICD10CM:R65.20	Severe sepsis without septic shock
Diagnosis	UMLS:ICD10CM:A20.7	Septicemic plague
Diagnosis	UMLS:ICD10CM:R65.21	Severe sepsis with septic shock
Diagnosis	UMLS:ICD10CM:R78.81	Bacteremia
**Pneumonia**		
**Outcome definition**		
Diagnosis	UMLS:ICD10CM:J13	Pneumonia due to Streptococcus pneumoniae
Diagnosis	UMLS:ICD10CM:J14	Pneumonia due to Hemophilus influenzae
Diagnosis	UMLS:ICD10CM:J15	Bacterial pneumonia, not elsewhere classified
Diagnosis	UMLS:ICD10CM:J16	Pneumonia due to other infectious organisms, not elsewhere classified
Diagnosis	UMLS:ICD10CM:J18	Pneumonia, unspecified organism
**Hospitalization**		
**Outcome definition**		
Visit	UMLS:HL7V3.0:VisitType:IMP	Visit: Inpatient Encounter
Visit	UMLS:HL7V3.0:VisitType:NONAC	Visit: Inpatient Non-acute
**ED visiting**		
**Outcome definition**		
Visit	UMLS:HL7V3.0:VisitType:EMER	Visit: Emergency
**Genital infection**		
**Outcome definition**		
Diagnosis	UMLS:ICD10CM:B37.3	Candidiasis of vulva and vagina
Diagnosis	UMLS:ICD10CM:B37.41	Candidal cystitis and urethritis
Diagnosis	UMLS:ICD10CM:B37.42	Candidal balanitis
Diagnosis	UMLS:ICD10CM:B37.49	Other urogenital candidiasis
Diagnosis	UMLS:ICD10CM:N34.1	Nonspecific urethritis
Diagnosis	UMLS:ICD10CM:N39.0	Urinary tract infection, site not specified
**Diabetic Ketoacidosis**		
**Outcome definition**		
Diagnosis	UMLS:ICD10CM:E11.1	Type 2 diabetes mellitus with ketoacidosis
Diagnosis	UMLS:ICD10CM:E13.1	Other specified diabetes mellitus with ketoacidosis
**Hypoglycemia**		
**Outcome definition**		
Diagnosis	UMLS:ICD10CM:E16.2	Hypoglycemia, unspecified

Diagnosis	UMLS:ICD10CM:E16.0	Drug-induced hypoglycemia without coma
Diagnosis	UMLS:ICD10CM:E16.1	Other hypoglycemia
Diagnosis	UMLS:ICD10CM:E16.2	Hypoglycemia, unspecified

3. Propensity Score Matching and baseline data

Propensity score matching was performed on all listed characteristics. Characteristics of the cohorts before and after matching are summarized in the table below.

Cohort 1 and cohort 2 patient count before and after propensity score matching

Cohort	Patient count before matching	Patient count after matching
1 - SGLT2i_ESRD_3m	5,988	5,295
2 - DPP4i_ESRD_3m	14,990	5,295

Propensity score density function - Before and after matching (cohort 1 - purple, cohort 2 - green)

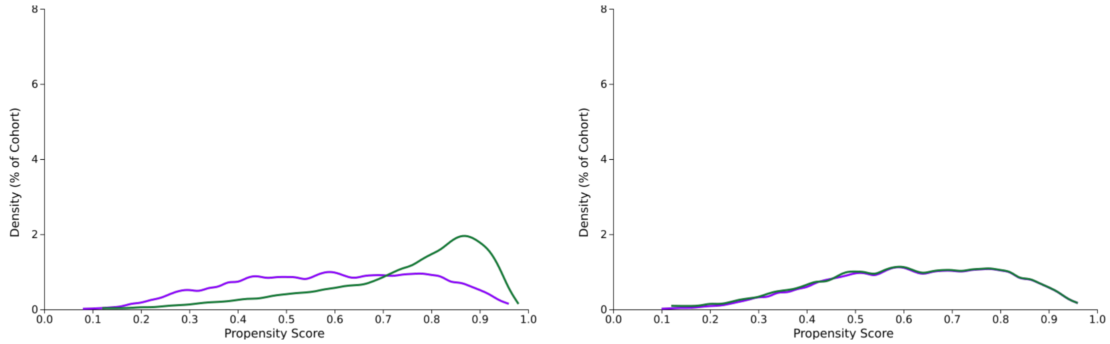

Cohort 1 (N = 5,988) and cohort 2 (N = 14,990) characteristics before propensity score matching

Demographics

AI	Age at Index
2106-3	White
2054-5	Black or African American
M	Male
2131-1	Other Race
2028-9	Asian

Diagnosis

nternational Classification of Diseases, Tenth Revision,	Name
E78	Disorders of lipoprotein metabolism and other lipidemias
I50	Heart failure
I10-I1A	Hypertensive diseases

I42	Cardiomyopathy			
I44	Atrioventricular and left bundle-branch block.			
I20-I25	Ischemic heart diseases			
I74	Arterial embolism and thrombosis			
I71	Aortic aneurysm and dissection			
I60-I69	Cerebrovascular diseases			
I26-I28	Pulmonary heart disease and diseases of pulmonary circulation			
I05-I09	Chronic rheumatic heart diseases			
E00-E07	Disorders of thyroid gland			
K74	Fibrosis and cirrhosis of liver			
F17	Nicotine dependence			
I70.2	Atherosclerosis of native arteries of the extremities			
M10	Gout			
F41	Other anxiety disorders			
K76.0	Fatty (change of) liver, not elsewhere classified			
I70.2	Atherosclerosis of native arteries of the extremities			
J44	Other chronic obstructive pulmonary disease			
**Medication**				
Anatomical Therapeutic Chemical	Name			
A10A	INSULINS AND ANALOGUES			
A10BB	Sulfonylureas			
A10BG	Thiazolidinediones			
C10AA	HMG CoA reductase inhibitors			
C10AB	Fibrates			
C09	AGENTS ACTING ON THE RENIN-ANGIOTENSIN SYSTEM			
C07	BETA BLOCKING AGENTS			
C08	CALCIUM CHANNEL BLOCKERS			
C04	PERIPHERAL VASODILATORS			
B01	ANTITHROMBOTIC AGENTS			
**Laboratory**				
Anatomical Therapeutic Chemical	Name	Unit	Missing rate after matching	
			SGLT2i users	DPP4i users
9029	Sodium in Serum	mmol/L	11.9%	19.7%
9028	Potassium in Serum	mmol/L	12.7%	20.5%
9030	Urea nitrogen Serum	mg/dL	13.2%	21.2%

9022	Calcium in Serum	mg/dL	12.9%	20.5%
9026	Magnesium in Serum	mg/dL	48.5%	55.5%
9027	Phosphate in Serum	mg/dL	41%	44.1%
9014	Hemoglobin in Blood	g/dL	15.6%	22.8%
9044	Alanine aminotransferase in Serum	U/L	17.6%	25.2%
9047	Aspartate aminotransferase in Serum	U/L	18.3%	25.4%
9046	Alkaline phosphatase in Serum	U/L	18.8%	25.5%
9045	Albumin in Serum	g/dL	16.3%	23.3%
9002	Cholesterol in LDL in Serum	mg/dL	34.4%	50%
9001	Cholesterol in HDL in Serum	mg/dL	32.8%	49%
9004	Triglyceride in Serum	mg/dL	32.8%	48.6%
9037	Hemoglobin A1c in Blood	%	22.6%	32.7%
9083	BMI	kg/m2	22.9%	27.7%
9024	Creatinine in Serum	mg/dL	12.4%	18.5%

Method S2. The detail of propensity score matching in TriNetX platform

(https://support.trinetx.com/hc/en-us/articles/360011978033)

To conduct PSM using the TNX Research platform, you must first identify two cohorts of interest, index events, outcomes of interest, and attributes of patients which may act as confounders to the outcomes of interest. For the purpose of this article, these attributes will be called "covariates."

Within Balance, when you run a propensity score matching analysis, the system conducts a propensity score matching to balance the cohorts:

1. For each patient in each cohort, the system computes values for each covariate.
2. These data form a matrix of covariate values for each
3. The system performs a logistic regression on the pooled matrices, to "predict" which cohort each patient originates from. The value of this model for a patient is that patient's predicted probability of being in the second cohort or "propensity score."
4. For each patient in the smaller cohort, the system chooses as match from the larger cohort (if any patients in the larger cohort are close enough). The pairs then form a subset of each cohort.

Within Outcomes, when you run compare cohorts after matching, the system conducts a propensity score matching to compare outcomes between the balance the cohorts:

1. For each patient in each cohort, the system computes both the outcome(s) of interest and values for each covariate.
2. These data form a matrix of covariate values for each
3. The system performs a logistic regression on the pooled matrices, to "predict" which cohort each patient originates from. The value of this model for a patient is that patient's predicted probability of being in the second cohort or "propensity score."
4. For each patient in the smaller cohort, the system chooses as match from the larger cohort (if any patients in the larger cohort are close enough). The pairs then form a subset of each cohort.
5. The system compares outcomes on these after matching subsets, rather than the original cohorts.

Values in the Covariate Matrix

In the covariate matrix, each row represents one patient, and each column represents one covariate. Each cell contains exactly one non-null numerical value.

All covariates are one of the following forms:

- **Binary:** yes/no;
- **Categorical:** real values placed in categories based on their value; or
- **Continuous:** real values represented directly.

When a covariate is binary (for example, a patient having history of asthma), the cell for that patient and covariate is set to 0 for “not present” or 1 for “is present.”

When a covariate is categorical, the categories are specified as ranges that the continuous variable can take (for example, having a value for a Sodium lab between 130 and 140). Each category becomes a distinct covariate which is either 0 for “not present” or 1 for “is present.” It is possible for all columns to be 0 (for example if there are no values in the time window, or if all values are outside all the categories). It is also possible for multiple values to be 1 (for example if there are multiple values in the time window, or if the categories overlap).

When a covariate is continuous (for example, current age, or age at time of the index event), then the value for that patient (if any) is placed in the relevant cell.

Details on Scoring and Logistic Regression

When you run propensity score matching, the system generates a propensity score for each patient in each cohort. The propensity score ranges between 0 and 1 and indicates the predicted probability a patient is in cohort B given the patient’s covariates.

To uses logistic regression to generate the propensity scores, through an implementation of the well-tested, standard software package scikit-learn. The code used in production is as follows (some logging, etc. removed for clarity):

```
import numpy as np

from sklearn.linear_model import LogisticRegression

from sklearn.preprocessing import Imputer

from impute import fill_nans

def fill_nans(data, axis=0):

    imp = Imputer(missing_values='NaN', strategy=method, axis=axis)

    return imp.fit_transform(data)

def propensity_scores(matrix_a, matrix_b):

    matrix_a = fill_nans(matrix_a, method='mean')

    matrix_b = fill_nans(matrix_b, method='mean')

    full_matrix = np.concatenate((matrix_a, matrix_b), axis=0)

    # cohort A is "zero" and cohort B is "one" from the regression’s perspective
```

```
    target_a = np.zeros(matrix_a.shape[0], dtype=float)

    target_b = np.ones(matrix_b.shape[0], dtype=float)

    target = np.concatenate([target_a, target_b])

    lr = LogisticRegression(C=1000)

    lr.fit(full_matrix, target)

    def get_scores(X):

        # .predict_proba returns [prob_class_zero, prob_class_one] for each row,

        # while the score we want is the probability of being in class B

        full_scores = lr.predict_proba(X)

        return full_scores[:, 1]

    return (get_scores(matrix_a), get_scores(matrix_b))
```

Here the "score" for each patient is the regression model applied to that patient's row. Informally, this is the probability that the patient belongs to cohort B, based on the cohorts it has seen.

Here "matrix_a" is the covariate matrix for the cohort you selected as Cohort A. Likewise, "matrix_b" is the covariate matrix for the cohort you selected as Cohort B. Data are pooled across all HCOs, so all patients in the analysis are a row in one of these matrices. Note that the code instructs the system to replace all missing (NaN) values in each matrix with the mean from that column; however, at time of writing all covariates are either binary, categorical (which expands to a set of binary columns), or continuous but guaranteed to exist, so this imputation is vacuous.

The call "LogisticRegression(C=1000)" means we predict the probability of being in cohort B using logistic regression. The value of C means we weight the mean residual by a factor of 1000 as compared to the norm of the coefficients. This means we are using a very small amount of L2 (ridge) regression, which is needed to make the objective function convex (so the regression converges), but with such a small value, it has very little effect on the model. Due to the very small amount of regularization and the scale of our covariates being constrained, it is not necessary to perform mean normalization as a preprocessing step.

Matching Details

Once the system has generated a propensity score for each patient, the system performs matching to identify the matched subsets. We use "greedy nearest neighbor matching" with a caliper of 0.1 pooled standard deviations. Sample code is here:

```
import numpy as np

def _pooled_sd(floats_a, floats_b):

    var_a = np.var(floats_a)

    var_b = np.var(floats_b)

    return sqrt((var_a + var_b) / 2.0)

def _naive_nearest_neighbor_match(scores_a, scores_b, caliper):

    # Perform nearest neighbor matching. Assume that len(scores_a) <=len(scores_b)

    num_a, num_b = len(scores_a), len(scores_b)

    max_diff = _pooled_sd(scores_a, scores_b) * caliper

    chosen_a = np.zeros(num_a, dtype=bool)  # True if the patient is matched, start false

    chosen_b = np.zeros(num_b, dtype=bool)

    for a_ind in range(0, num_a):

        best_diff = max_diff

        best_b_ind = None

        found_any = False

        for b_ind in range(0, num_b):

            if chosen_b[b_ind]:

                continue

            diff = abs(scores_a[a_ind] - scores_b[b_ind])

             if diff < best_diff or (diff == best_diff and not found_any):

                best_diff = diff

                best_b_ind = b_ind

                found_any = True
```

```
        if best_b_ind is not None:
            chosen_a[a_ind] = True
            chosen_b[best_b_ind] = True
    return (chosen_a, chosen_b)
```

Note: the code used in production is highly optimized and significantly longer. The above code is taken directly from our test fixtures and is used to ensure that the optimized code produces identical results to the above simplified code.

We use a "caliper" of 0.1 pooled standard deviations of the propensity scores in aggregate, which means that patients with very different propensity scores are not matched. The system matches patients using the following algorithm:

1. For each patient in cohort A (assumed to be the smaller of the two), and identify the patient whose score is closest to the patient in cohort A from the patients in cohort B who have not yet been matched.
2. If a match is found, mark both as "chosen" and move on.
3. At the end, return the labels of each cohort, indicating which patients were chosen and which were not. The patients who were chosen form the "matched" cohorts.

Pooling the Cohorts, and Compensating for Unbalanced Cohorts across HCOs

TriNetX pulls data from a federated data network made up of many healthcare organizations across the world. Each site computes a covariate matrix for the patients they contribute to the analysis and send it to a central processing point to be pooled and analyzed as a single matrix.

The order of the rows in the matrix should not impact the propensity scores generated for each patient; logistic regression is highly stable with respect to permuting the rows. In contrast, nearest neighbor matching, can be influenced by the order of rows in the matrix. For example, if two identical patients in cohort B match equally well to a patient in cohort A, the first will be chosen and the second will not. Therefore, if the order of the rows carries some information (perhaps as an artifact of the pooling), the order can introduce bias.

To eliminate this bias, we randomize the order of the records in the covariate matrix. We sort the provider results by the unique ID of the HCO (so the information of "which HCO responded first" is deleted), concatenate the matrices into one large matrix, then shuffle them using `np.random.shuffle(full_matrix_a)` and `np.random.shuffle(full_matrix_b)`. To assure determinism, a call to `np.random.seed(FIXED_SEED)` precedes all calls to shuffle, so that successive runs do not change unless the underlying data changes.

Figure S1. Schematic of study design

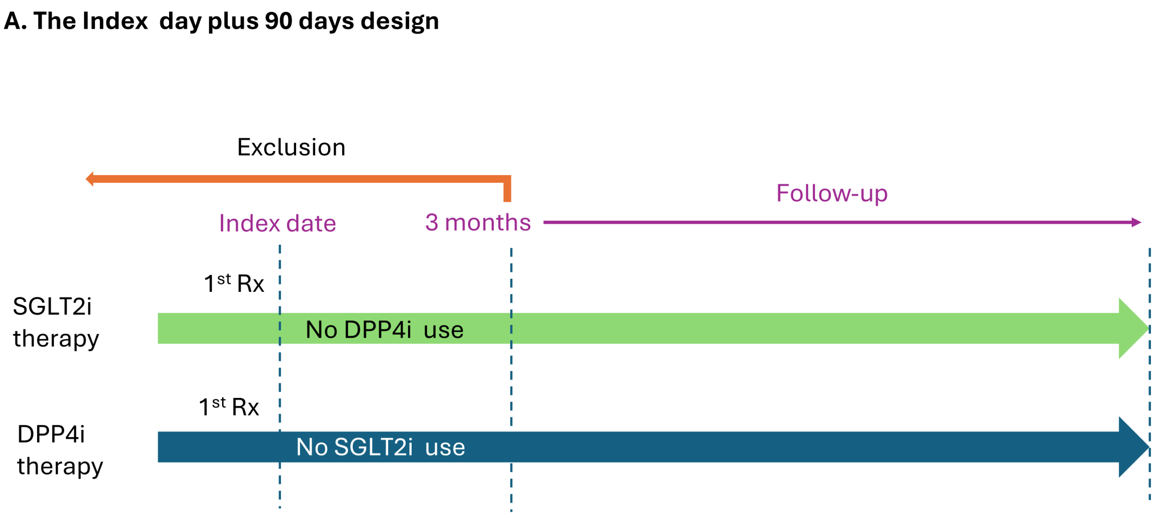

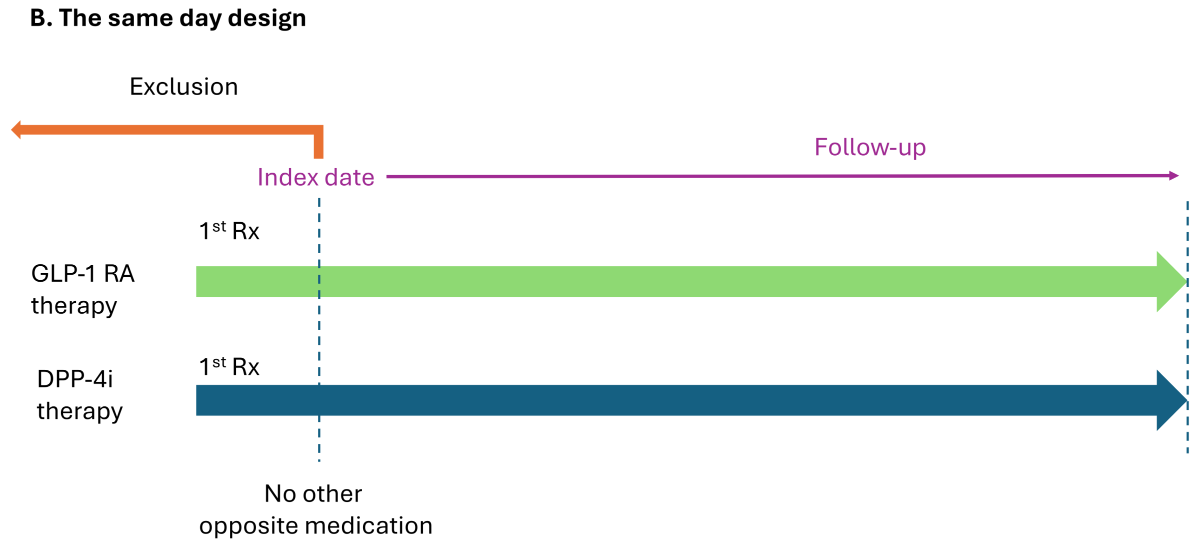

Figure S2. Time distribution of studied cohorts

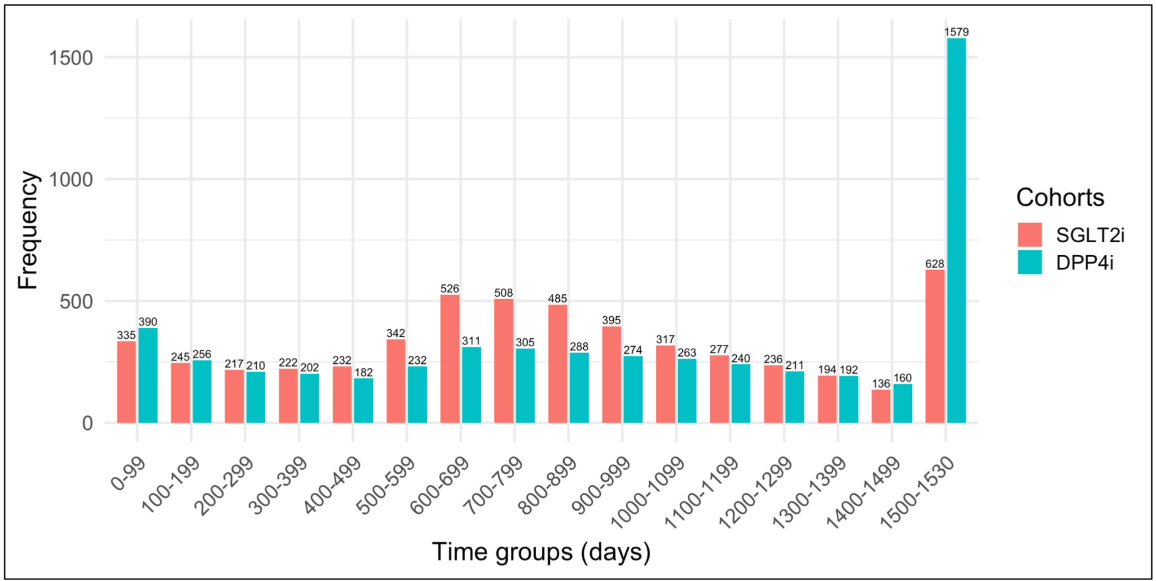

Follow-up Time (After Matching)

Cohort	Mean Follow-up (Days)	Standard Deviation	Median Follow-up (Days)	Interquartile Range
SGLT2i	816.938	441.947	800	618
DPP4i	949.887	519.767	994	998

Figure S3. KM for other clinical outcomes

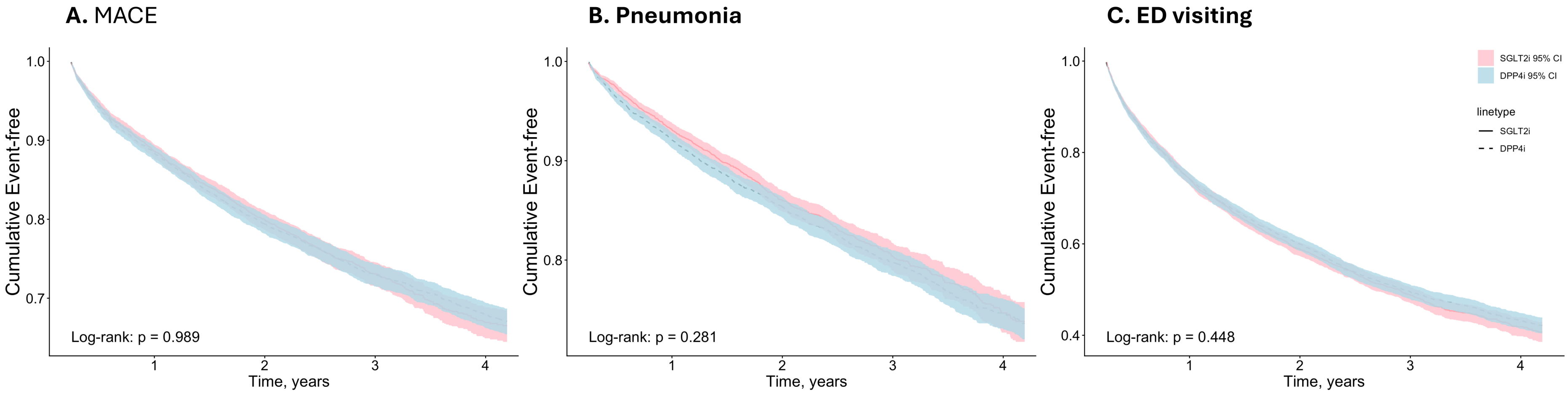

Figure S4. Subgroup analysis for mortality

Subgroup	SGLT2i events (%)	DPP4i events (%)	HR (95%CI)	p value	Heterogeneity p value
**Sex**					**0.78**
Female	287 (24.11)	367 (24.23)	0.90 (0.77 - 1.06)	0.202	
Male	458 (25.73)	594 (28.31)	0.93 (0.82 - 1.05)	0.244	
**Age (years)**					**0.938**
≥65	376 (22.24)	490 (23.83)	0.94 (0.82 - 1.08)	0.404	
<65	413 (27.08)	518 (29.00)	0.94 (0.82 - 1.07)	0.326	
**Race**					**0.044**
Asian	32 (18.24)	50 (23.78)	0.79 (0.51 - 1.24)	0.313	
African American	116 (18.91)	158 (22.62)	0.86 (0.68 - 1.09)	0.219	
White	401 (26.73)	564 (25.72)	1.11 (0.98 - 1.27)	0.117	
**BMI (kg/m²)**					**0.867**
≥30	204 (29.52)	257 (32.46)	0.94 (0.78 - 1.13)	0.498	
<30	215 (24.50)	267 (24.05)	0.96 (0.80 - 1.15)	0.649	
**HbA1c(%)**					**0.107**
≥7	250 (26.96)	304 (27.59)	0.94 (0.80 - 1.11)	0.483	
<7	224 (20.38)	322 (26.51)	0.77 (0.65 - 0.92)	0.003	
**Cerebrovascular diease**					**0.468**
Yes	241 (34.18)	305 (37.67)	0.90 (0.76 - 1.06)	0.211	
No	452 (21.72)	547 (22.57)	0.97 (0.86 - 1.10)	0.636	
**Heart failure**					**0.271**
Yes	531 (33.26)	691 (37.66)	0.87 (0.78 - 0.98)	0.021	
No	152 (14.80)	182 (14.27)	1.00 (0.81 - 1.25)	0.98	
**Ischemic heart disease**					**0.586**
Yes	580 (31.20)	764 (35.38)	0.87 (0.78 - 0.97)	0.01	
No	172 (16.25)	219 (16.91)	0.92 (0.76 - 1.13)	0.444	
**Acute myocardial infarction**					**0.43**
Yes	219 (42.04)	285 (47.92)	0.82 (0.69 - 0.98)	0.032	
No	528 (21.05)	695 (23.40)	0.90 (0.80 - 1.01)	0.062	

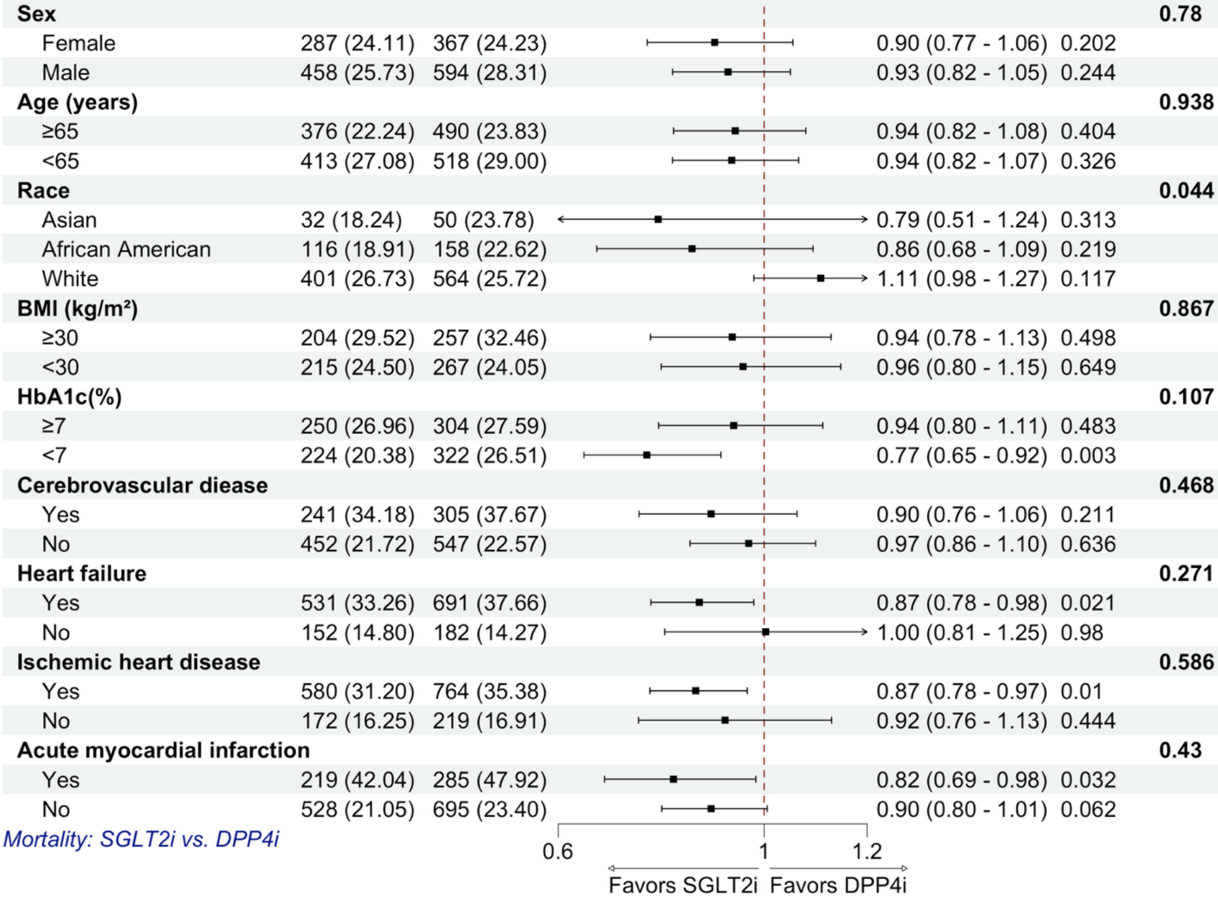

Mortality: SGLT2i vs. DPP4i

Figure S5. Subgroup analysis for sepsis

Subgroup	SGLT2i events (%)	DPP4i events (%)	HR (95%CI)	p value	Heterogeneity p value
**Sex**					**1**
Female	333 (27.14)	416 (28.12)	0.88 (0.76 - 1.02)	0.083	
Male	524 (28.14)	677 (32.96)	0.88 (0.78 - 0.99)	0.029	
**Age (years)**					**0.22**
≥65	492 (27.26)	652 (30.87)	0.85 (0.76 - 0.96)	0.009	
< 65	416 (27.36)	492 (28.59)	0.95 (0.84 - 1.09)	0.49	
**Race**					**0.863**
Asian	43 (22.25)	54 (25.75)	0.93 (0.62 - 1.39)	0.706	
African American	164 (26.58)	227 (33.35)	0.83 (0.67 - 1.01)	0.063	
White	416(27.06)	678(31.35)	0.87 (0.75 - 1.01)	0.67	
**BMI (kg/m²)**					**0.564**
≥30	176 (25.91)	233 (29.54)	0.82 (0.68 - 1.00)	0.052	
<30	251 (26.12)	316 (29.44)	0.89 (0.75 - 1.05)	0.162	
**HbA1c(%)**					**0.271**
≥7	254 (26.22)	356 (32.64)	0.76 (0.64 - 0.89)	0.001	
<7	273 (25.72)	341 (28.66)	0.86 (0.73 - 1.01)	0.064	
**Cerebrovascular diease**					**0.949**
Yes	261 (37.05)	307 (37.91)	0.90 (0.76 - 1.06)	0.225	
No	490 (23.40)	614 (25.93)	0.90 (0.80 - 1.01)	0.071	
**Heart failure**					**0.978**
Yes	610 (37.49)	705 (39.92)	0.93 (0.83 - 1.04)	0.203	
No	179 (16.67)	217 (16.66)	0.94 (0.77 - 1.14)	0.511	
**Ischemic heart disease**					**0.597**
Yes	658 (34.94)	781 (37.10)	0.92 (0.83 - 1.02)	0.119	
No	186 (16.35)	242 (19.10)	0.87 (0.72 - 1.05)	0.151	
**Acute myocardial infarction**					**0.729**
Yes	236 (43.43)	262 (42.81)	0.89 (0.74 - 1.06)	0.175	
No	595 (23.33)	737 (25.65)	0.92 (0.82 - 1.02)	0.125	

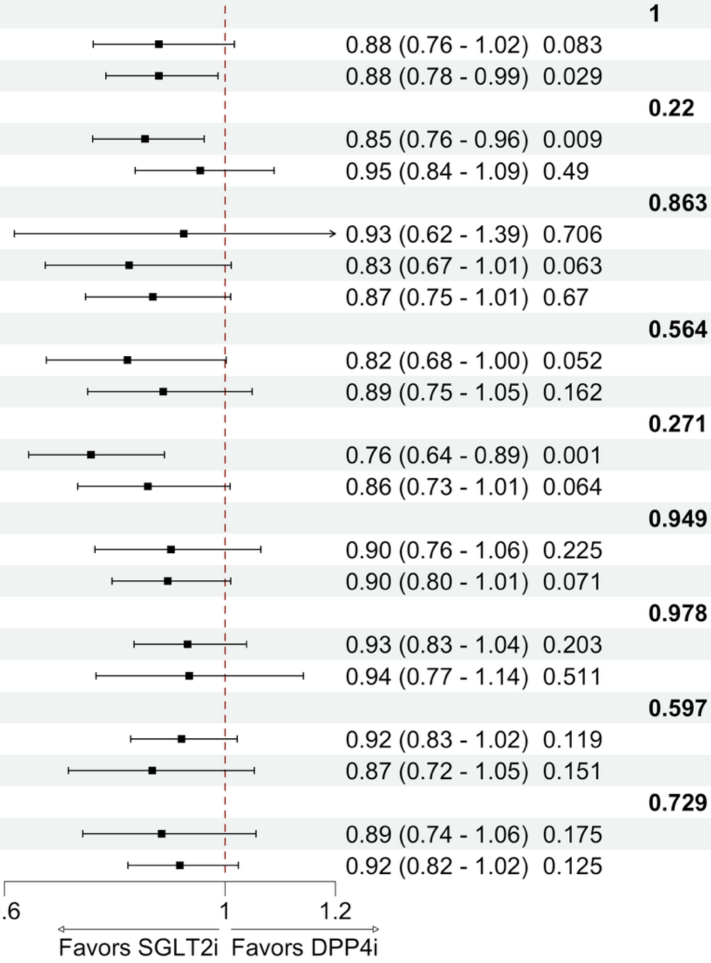

Sepsis: SGLT2i vs. DPP4i

Figure S6. Subgroup analysis for hospitalization

Subgroup	SGLT2i events (%)	DPP4i events (%)	HR (95%CI)	p value	Heterogeneity p value
**Sex**					**0.41**
Female	978 (66.82)	1.018 (63.18)	0.99 (0.90 - 1.08)	0.768	
Male	1,374 (65.65)	1,531 (66.58)	0.94 (0.87 - 1.01)	0.1	
**Age (years)**					**0.47**
≥65	1,405 (66.83)	1,565 (67.40)	0.95 (0.89 - 1.02)	0.183	
<65	1,181 (65.43)	1,320 (68.15)	0.92 (0.85 - 0.99)	0.027	
**Race**					**0.08**
Asian	111 (56.91)	142 (62.89)	0.85 (0.66 - 1.09)	0.199	
African American	529 (71.41)	550 (70.19)	1.00 (0.88 - 1.12)	0.95	
White	1,190 (65.50)	1,627 (70.27)	0.88 (0.82 - 0.95)	0.001	
**BMI (kg/m²)**					**0.72**
≥30	494 (62.14)	578 (66.40)	0.85 (0.75 - 0.95)	0.006	
<30	684 (60.89)	771 (64.05)	0.87 (0.79 - 0.97)	0.009	
**HbA1c(%)**					**0.77**
≥7	756 (65.56)	832 (69.11)	0.91 (0.83 - 1.01)	0.07	
<7	821 (64.87)	877 (65.33)	0.93 (0.85 - 1.02)	0.145	
**Cerebrovascular diease**					**0.27**
Yes	612 (71.43)	662 (74.03)	0.91 (0.82 - 1.02)	0.097	
No	1,463 (61.58)	1,573 (59.73)	0.98 (0.91 - 1.05)	0.6	
**Heart failure**					**0.13**
Yes	1,500 (76.49)	1,594 (76.83)	0.92 (0.85 - 0.98)	0.014	
No	664 (52.59)	716 (52.43)	1.01 (0.91 - 1.12)	0.854	
**Ischemic heart disease**					**0.66**
Yes	1,658 (73.50)	1,746 (72.83)	0.97 (0.90 - 1.03)	0.322	
No	662 (53.45)	729 (53.06)	0.99 (0.90 - 1.11)	0.926	
**Acute myocardial infarction**					**0.93**
Yes	573 (85.39)	544 (78.35)	0.97 (0.86 - 1.09)	0.569	
No	1,753 (61.32)	1,943 (60.47)	0.96 (0.90 - 1.02)	0.222	

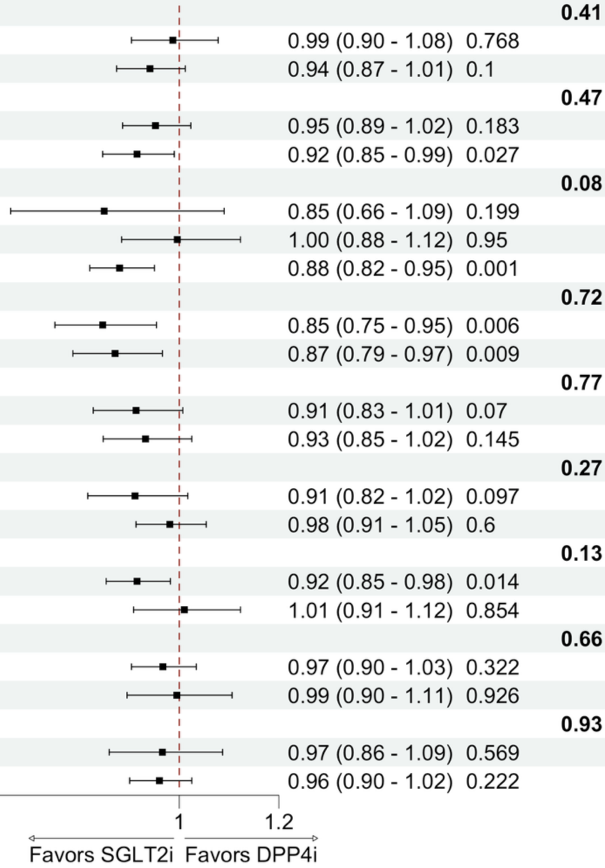

Hospitalization: SGLT2i vs. DPP4i

**Table S1**. Target trial emulation		
**Approach**	**Target Trial**	**Target Trial Emulation**
**Eligibility criteria**	Patients with type 2 diabetes mellitus (T2DM) and end-stage kidney disease (ESKD); adults (≥18 years); eligible for SGLT2 inhibitor therapy compared with an active comparator drug.	Adult patients (≥18 years) with T2DM and ESKD (ICD-10-CM code N18.6) identified from TriNetX US Collaborative Network (2016–2023). Excluded: ADPKD, vasculitis, organ transplant, recent immunosuppressant use, recent malignancy or liver dysfunction, recent MI or stroke, lupus nephritis, pregnancy, prior SGLT2i/DPP4i use.
**Treatment strategies**	Initiate SGLT2 inhibitor (canagliflozin, dapagliflozin, empagliflozin, ertugliflozin, bexagliflozin, sotagliflozin). Comparator group receives a clinically appropriate alternative antidiabetic agent (DPP4 inhibitor).	New-user, active-comparator design; first prescription of SGLT2i vs DPP4i. Index date = first prescription + 90 days. Follow-up from one day after index date.
**Treatment assignment**	Randomized assignment to SGLT2i vs comparator.	Nonrandomized; new-user cohort design with *propensity score matching (1:1)* based on demographic, clinical, medication, and laboratory covariates to emulate randomization.
**Outcomes**	Primary: all-cause mortality. Secondary: sepsis, pneumonia, major adverse cardiovascular events (MACE), all-cause hospitalization, emergency department (ED) admission.	Extracted from linked EHR data (TriNetX). Primary = mortality. Secondary = sepsis, pneumonia, MACE, hospitalization, ED admission.
**Follow-up**	From treatment initiation until outcome, loss to follow-up, or 4 years post-index. Censoring at death or end of study period.	Outcomes assessed from 1 day to 4 years after index date; censored at death, loss to follow-up, or study end date (October 4, 2025).
**Causal contrasts**	Intention-to-treat (ITT): effect of initial assignment to SGLT2i vs comparator on clinical outcomes.	ITT analysis comparing initial new users of SGLT2i vs DPP4i; each patient classified by initial drug class irrespective of subsequent changes/discontinuation.
**Statistical analysis**	Estimate hazard ratios for all-cause mortality and secondary outcomes using Cox proportional hazards models; use matching or adjustment for baseline covariates. Perform sensitivity analyses.	Cox proportional hazards model for outcome comparison; Kaplan–Meier survival curves. Sensitivity analyses tested alternate PSM models, time windows (1–4 years), definitions of ESKD, and omission of 3-month lag. E-

		values computed to assess robustness to unmeasured confounding.

Table S2. Hierarchical propensity score matching models

Clinical Outcomes	After propensity score matching with a follow-up of 4 years					
	Model 1		Model 2		Model 3	
	HR (95%CI)	*P*	HR (95%CI)	*P*	HR (95%CI)	*P*
**Primary outcomes**						
Mortality	0.82 (0.74–0.90)	<0.001	0.77 (0.69–0.86)	<0.001	0.80 (0.72–0.86)	<0.001
**Secondary outcomes**						
Sepsis	0.87 (0.79–086)	0.005	0.82 (0.74–0.91)	<0.001	0.83 (0.75–0.91)	<0.001
Pneumonia	1.00 (0.90–1.11)	0.966	0.92 (0.82–1.03)	0.134	0.94 (0.85–1.05)	0.271
MACE[#]	1.03 (0.94–1.13)	0.56	0.91 (0.83–1.01)	0.068	0.93 (0.85–1.01)	0.099[†]
Hospitalization	0.96 (0.91–1.02)	0.203 [†]	0.89 (0.83–0.94)	<0.001	0.92 (0.87–0.98)	0.009[†]
ED visiting	1.08 (1.01–1.151)	0.023	0.94 (0.88–1.01)	0.073[†]	1.02 (0.95–1.09)	0.635[†]

Model 1 adjusts for age, sex, race, and body mass index in propensity score matching. Model 2 builds on Model 1 by adding baseline comorbidities, while Model 3 further includes baseline medication use.

[†]This indicate the proportional hazard assumption is violated.

[#]MACE included the acute myocardial infarction, heart failure, stroke, and death.

Abbreviation: SGLT2i, glucagon-like peptide-1 receptor agonists ; DPP4i, sodium-glucose cotransporter-2 inhibitor ; HR, hazard ratio ; CI, confidence interval; MACE, major adverse cardiovascular events; ED, emergency department.

Table S3. Different time frames analysis

Clinical Outcomes	After propensity score matching[%], SGLT2i vs. DPP4i					
	3 moths to 1 year		3 moths to 2 years		3 moths to 3 years	
	HR (95%CI)	*P* value	HR (95%CI)	*P* value	HR (95%CI)	*P* value
**Primary outcomes**						
Mortality	0.91 (0.77–1.06)	0.215	0.89 (0.80–1.00)	0.044	0.90 (0.82–0.99)	0.038
**Secondary outcomes**						
Sepsis	0.87 (0.76–1.00)	0.042	0.85 (0.77–0.94)	0.001	0.86 (0.79–0.94)	0.001
Pneumonia	0.80 (0.69–0.84)	0.005	0.85 (0.76–0.95)	0.004	0.94 (0.85–1.04)	0.246
MACE[#]	0.97 (0.86–1.09)	0.615	0.95 (0.87–1.05)	0.305	0.99 (0.91–1.08)	0.863
Hospitalization	0.90 (0.84–0.97)	0.005[†]	0.93 (0.87–0.99)	0.017	0.92 (0.87–0.97)	0.002
ED visiting	0.994 (0.92–1.08)	0.885	1.03 (0.97–1.10)	0.372	1.02 (0.96–1.09)	0.490

[%]Propensity score matching used all the baseline variable in Table 1 of main article

[#]MACE included the acute myocardial infarction, heart failure, stroke, and death.

[†]This indicate the proportional hazard assumption is violated.

Abbreviation: SGLT2i, sodium-glucose cotransporter-2 inhibitor ; DPP4i, dipeptidyl peptidase-4 inhibitors ;HR, hazard ratio ; CI, confidence interval, MACE, major adverse cardiovascular events; ED, emergency department

Table S4. Clinical outcomes analysis without time lag post-index

Clinical Outcomes	After propensity score matching[%] with a follow-up of 4 years					
	SGLT2i users (n = 5,465)		DPP4i users (n = 5,465)		SGLT2i vs. DPP4i	
	Events (n)	Cumulative incidence (%)	Events (n)	Cumulative incidence (%)	HR (95%CI)	*P value*
**Primary outcomes**						
Mortality	1,022	26.91	1,327	30.46	0.86 (0.79–0.93)	<0.001
**Secondary outcomes**						
Sepsis	1,166	29.95	1,446	34.02	0.86 (0.80–0.93)	<0.001
Pneumonia	978	27.27	1,163	28.41	0.91 (0.84–0.99)	0.031
MACE[#]	1,328	35.25	1,480	34.95	0.97 (0.90–1.04)	0.364
Hospitalization	2,871	67.38	3,206	69.37	0.90 (0.86–0.95)	<0.001
ED visiting	2,434	60.29	2,515	58.58	1.03 (0.97–1.09)	0.356

[%]Propensity score matching used all the baseline variable in Table 1 of main article

[#]MACE included the acute myocardial infarction, heart failure, stroke, and death.

[†]This indicate the proportional hazard assumption is violated.

Abbreviation: SGLT2i, sodium-glucose cotransporter-2 inhibitor ; DPP4i, dipeptidyl peptidase-4 inhibitors ;HR, hazard ratio ; CI, confidence interval, MACE, major adverse cardiovascular events; ED, emergency department

Table S5. Outcomes in the different types of end stage of kidney disease

Clinical Outcomes	After propensity score matching[%], SGLT2i vs. DPP4i					
	eGFR < 15 ml/min/1.73 m^2 (n = 5,029 pairs)		eGFR < 10 ml/min/1.73 m^2 (n = 3,940 pairs)		Dialysis dependence (n = 2,773 pairs)	
	HR (95%CI)	*P* value	HR (95%CI)	*P* value	HR (95%CI)	*P* value
**Primary outcomes**						
Mortality	0.77 (0.68–0.86)	<0.001	0.61 (0.53–0.70)	<0.001	0.86 (0.76–0.98)	0.018
**Secondary outcomes**						
Sepsis	0.82 (0.73–0.93)	0.001	0.77 (0.66–0.90)	0.001	0.87 (0.77–0.97)	0.012
Pneumonia	0.79 (0.69–0.90)	<0.001	0.81 (0.69–0.96)	0.015	0.94 (0.83–1.06)	0.297
MACE[#]	0.84 (0.76–0.94)	0.002	0.82 (0.72–0.94)	0.005	0.98 (0.88–1.09)	0.682
Hospitalization	0.83 (0.78–0.90)	<0.001	0.85 (0.77–0.93)	<0.001	0.92 (0.86–0.99)	0.039[†]
ED visiting	0.87 (0.80–0.93)	<0.001[†]	0.93 (0.85–1.01)	0.088[†]	0.95 (0.88–1.03)	0.211

[%]Propensity score matching used all the baseline variable in Table 1 of main article

[#]MACE included the acute myocardial infarction, heart failure, stroke, and death.

[†]This indicate the proportional hazard assumption is violated.

Abbreviation: SGLT2i, sodium-glucose cotransporter-2 inhibitor ; DPP4i, dipeptidyl peptidase-4 inhibitors ;HR, hazard ratio ; CI, confidence interval, MACE, major adverse cardiovascular events; ED, emergency department

Table S6. Clinical outcomes analysis using 2000–2023 data

Clinical Outcomes	After propensity score matching[%] with a follow-up of 4 years					
	SGLT2i users (n = 2,665)		DPP4i users (n = 2,665)		SGLT2i vs. DPP4i	
	Events (n)	Cumulative incidence (%)	Events (n)	Cumulative incidence (%)	HR (95%CI)	*P value*
**Primary outcomes**						
Mortality	374	21.6	595	27.5	0.83 (0.73–0.94)	0.004[†]
**Secondary outcomes**						
Sepsis	439	24.7	818	38.4	0.64 (0.57–0.71)	<0.001
Pneumonia	400	22.7	654	31.6	0.75 (0.66–0.85)	<0.001
MACE[#]	560	32.7	796	37.1	0.86 (0.77–0.96)	0.006
Hospitalization	1,309	63.9	1,775	66.5	0.77 (0.71–0.82)	<0.001[†]
ED visiting	1,175	60	1,557	68.9	0.81 (0.75–0.88)	<0.001

[%]Propensity score matching used all the baseline variable in Table 1 of main article

[#]MACE included the acute myocardial infarction, heart failure, stroke, and death.

[†]This indicate the proportional hazard assumption is violated.

Abbreviation: SGLT2i, sodium-glucose cotransporter-2 inhibitor ; DPP4i, dipeptidyl peptidase-4 inhibitors ;HR, hazard ratio ; CI, confidence interval, MACE, major adverse cardiovascular events; ED, emergency department

Table S7. Clinical outcomes analysis excluding the death in lag period

Clinical Outcomes	After propensity score matching% with a follow-up of 4 years					
	SGLT2i users (n = 4,648)		DPP4i users (n = 4,648)		SGLT2i vs. DPP4i	
	Events (n)	Cumulative incidence (%)	Events (n)	Cumulative incidence (%)	HR (95%CI)	*P value*
**Primary outcomes**						
Mortality	766	23.7	973	27.8	0.87 (0.79–0.96)	0.003
**Secondary outcomes**						
Sepsis	830	25.8	1.058	30.9	0.83 (0.75–0.91)	<0.001
Pneumonia	782	25.2	951	27.8	0.88 (0.79–0.95)	0.003†
MACE#	1,061	33.2	1,157	33.6	0.98 (0.91–1.01)	0.714
Hospitalization	2,392	66.7	2,607	68.9	0.91 (0.86–0.96)	<0.001
ED visiting	2,149	61.2	2,294	62.8	0.96 (0.90–1.01)	0.129

%Propensity score matching used all the baseline variable in Table 1 of main article

#MACE included the acute myocardial infarction, heart failure, stroke, and death.

†This indicate the proportional hazard assumption is violated.

Abbreviation: SGLT2i, sodium-glucose cotransporter-2 inhibitor ; DPP4i, dipeptidyl peptidase-4 inhibitors ;HR, hazard ratio ; CI, confidence interval, MACE, major adverse cardiovascular events; ED, emergency department

Table S8. Clinical outcomes analysis including more anti-diabetics medications

Clinical Outcomes	After propensity score matching[%] with a follow-up of 4 years					
	SGLT2i users (n = 3,782)		DPP4i users (n = 3,782)		SGLT2i vs. DPP4i	
	Events (n)	Cumulative incidence (%)	Events (n)	Cumulative incidence (%)	HR (95%CI)	*P value*
**Primary outcomes**						
Mortality	603	23.7	752	27	0.87 (0.78–0.97)	0.011
**Secondary outcomes**						
Sepsis	643	25.7	779	29	0.87 (0.78–0.96)	0.008
Pneumonia	592	24.7	718	27	0.87 (0.78–0.97)	0.009
MACE[#]	851	34.2	909	32.8	0.99 (0.90–1.09)	0.887
Hospitalization	1,846	65.2	1,967	66.7	0.95 (0.89–1.01)	0.084
ED visiting	1,671	60.6	1,783	61.7	0.96 (0.89–1.02)	0.174

[%]Propensity score matching used all the baseline variable in Table 1 of main article and adding the medication of glucagon-like peptide-1 receptor agonists and repaglinide.

[#]MACE included the acute myocardial infarction, heart failure, stroke, and death.

[†]This indicate the proportional hazard assumption is violated.

Abbreviation: SGLT2i, sodium-glucose cotransporter-2 inhibitor ; DPP4i, dipeptidyl peptidase-4 inhibitors ;HR, hazard ratio ; CI, confidence interval, MACE, major adverse cardiovascular events; ED, emergency department

Table S9. Side effects analysis in the patients with types 2 DM and ESKD

Clinical O8utcomes	After propensity score matching[%] with a follow-up of 4 years					
	SGLT2i users (n = 4,835)		DPP4i users (n = 4,835)		SGLT2i vs. DPP4i	
	Events (n)	Cumulative incidence (%)	Events (n)	Cumulative incidence (%)	HR (95%CI)	*P value*
Genital tract infection	382	18.2	407	17.7	1.04 (0.90–1.20)	0.612
Diabetic ketoacidosis	78	3.1	71	2.5	1.26 (0.91–1.74)	0.163
Hypoglycemia	336	11.8	362	11.4	1.03 (0.89–1.20)	0.694

[%]Propensity score matching used all the baseline variable in Table 1 of main article

[†]This indicate the proportional hazard assumption is violated.

Abbreviation: SGLT2i, sodium-glucose cotransporter-2 inhibitor ; DPP4i, dipeptidyl peptidase-4 inhibitors ;HR, hazard ratio ; CI, confidence interval.
